# Screening of candidate analgesics using a patient‐derived human iPSC model of nociception identifies putative compounds for therapeutic treatment

**DOI:** 10.1002/ctm2.70339

**Published:** 2025-05-25

**Authors:** Jack R. Thornton, Alberto Capurro, Sally Harwood, Thomas C Henderson, Adrienne Unsworth, Franziska Görtler, Sushma Nagaraja‐Grellscheid, Vsevolod Telezhkin, Majlinda Lako, Evelyne Sernagor, Lyle Armstrong

**Affiliations:** ^1^ Biosciences Institute Newcastle University Newcastle‐upon‐Tyne UK; ^2^ Bioinformatics Support Unit Newcastle University Newcastle‐upon‐Tyne UK; ^3^ Department of Biological Sciences University of Bergen Bergen Norway; ^4^ School of Dental Sciences Newcastle University Newcastle‐upon‐Tyne UK; ^5^ Present address: Alberto Capurro Queen Mary University of London Centre of Neuroscience, Surgery and Trauma The Blizard Institute London UK

**Keywords:** analgesic candidates, drug screening, electrophysiology, induced pluripotent stem cells (iPSCs), inherited erythromelalgia (IEM), sensory neuron

## Abstract

**Background and purpose:**

In this study, we applied an induced pluripotent stem cell (iPSC)‐based model of inherited erythromelalgia (IEM) to screen a library of 281 small molecules, aiming to identify candidate pain‐modulating compounds.

**Experimental approach:**

Human iPSC‐derived sensory neuron‐like cells, which exhibit action potentials in response to noxious stimulation, were evaluated using whole‐cell patch‐clamp and microelectrode array (MEA) techniques.

**Key results:**

Sensory neuron‐like cells derived from individuals with IEM showed spontaneous electrical activity characteristic of genetic pain disorders. The drug screen identified four compounds (AZ106, AZ129, AZ037 and AZ237) that significantly decreased spontaneous firing with minimal toxicity. The calculated IC_50_ values indicate the potential efficacy of these compounds. Electrophysiological analysis confirmed the compounds’ ability to reduce action potential generation in IEM patient‐specific iPSC‐derived sensory neuron‐like cells.

**Conclusions and implications:**

Our screening approach demonstrates the reproducibility and effectiveness of human neuronal disease modelling offering a promising avenue for discovering new analgesics. These findings address a critical gap in current therapeutic strategies for both general and neuropathic pain, warranting further investigation. This study highlights the innovative use of patient‐derived iPSC sensory neuronal models in pain research and emphasises the potential for personalised medicine in developing targeted analgesics.

**Key points:**

- Utilisation of human iPSCs for efficient differentiation into sensory neuron‐like cells offers a novel strategy for studying pain mechanisms.
- IEM sensory neuron‐like cells exhibit key biomarkers and generate action potentials in response to noxious stimulation.
- IEM sensory neuron‐like cells display spontaneous electrical activity, providing a relevant nociceptive model.
- Screening of 281 compounds identified four candidates that significantly reduced spontaneous firing with low cytotoxicity.
- Electrophysiological profiling of selected compounds revealed promising insights into their mechanisms of action, specifically modulating the Na_V_ 1.7 channel for targeted analgesia.

## Graphical abstract

In this study, Thornton and colleagues utilised an induced pluripotent stem cell (iPSC)‐based model of inherited erythromelalgia (IEM) to screen a library of 295 small molecules in search of potential pain‐modulating compounds. Their screening identified four compounds that significantly reduced spontaneous firing in iPSC‐derived nociceptor‐like cells, with minimal associated toxicity

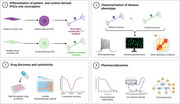

## 1 INTRODUCTION

Chronic pain is a global healthcare problem affecting approximately 30% of adults, with a higher prevalence among women.^1^ In the UK alone, an estimated 14 million people suffer from chronic pain, defined as pain lasting more than 3 months. A quarter of these individuals report that their pain significantly impairs their social functioning, including missing more than 2 weeks of work within 3 months. Globally, the prevalence of chronic pain ranges from 11.5 to 55%, and this is anticipated to rise due to ageing populations and increasing incidences of pain‐causing conditions.^2^ The socio‐economic burden of chronic pain is substantial, characterised by lost work hours and increased frequency of healthcare visits. In Canada, direct healthcare costs associated with chronic pain were estimated to exceed $6 billion annually, with productivity losses calculated at $37 billion per year.^3^ In the USA, the combined direct and indirect costs are approximately $100 billion annually.^4^ Despite this immense human and economic toll and considerable pharmaceutical investment, current therapies are limited by their effectiveness and adverse side effects. Common treatments, although somewhat effective, often lead to issues such as addiction and gastrointestinal complications, limiting their long‐term viability.^5^ Nearly half of European adults with moderate to severe chronic pain receive inadequate pain management, and only 2% report assistance from a pain specialist.^6^

There is a critical, unmet need for the development of more precise and effective pain management medications. Progress in new analgesic therapeutics has been hindered by the lack of effective mechanisms for translating pre‐clinical research to clinical settings. Much of the existing research relies on animal models, which frequently fail to accurately recapitulate human physiological responses to pain‐evoking stimuli.^7^

Chronic pain is a multifactorial disorder with poorly understood pathophysiology. Pain perception relies on nociceptive neurons detecting noxious stimuli and involves two primary processes: the generation of a sensor potential via a noxious stimulus (i.e., heat, cold, mechanical pressure or chemical mediators) and the translation of this sensor potential into an action potential. In chronic pain, neural dysfunction can cause hyperactivity (hyperalgesia) and spontaneous action potential (sAP) generation (allodynia) by nociceptive neurons. Voltage‐gated sodium channels (VGSCs) are crucial for the electrical activity of cells, playing a key role in action potential generation and propagation.^8^ Changes in VGSC function can significantly impact neuronal excitability and pain signalling, making these channels promising targets for new analgesics.^9^

VGSC type 1.7 (Na_V_ 1.7), encoded by the *SCN9A* gene, stands out as a particularly promising target. Na_V_ 1.7 determines the threshold for action potential generation in nociceptive neurons, and strong genetic evidence supports its critical role in pain transmission. Individuals with loss‐of‐function mutations in *SCN9A* exhibit congenital insensitivity to pain, while gain‐of‐function mutations cause chronic pain conditions such as paroxysmal extreme pain disorder and inherited erythromelalgia (IEM). Several compounds targeting Na_V_ 1.7 have been developed, with two showing efficacy in small cohorts of IEM patients.^10^, ^11^ However, many Na_V_ 1.7 specific compounds have failed in clinical trials due to modest efficacy against neuropathic pain, or lack of activity against other pain mechanisms.^12^ A major issue with these molecules is the lack of selectivity for the Na_V_ 1.7 subunit.

Alternative targets have yielded some effective therapeutic molecules for neuropathic pain, such as gabapentin and pregabalin, which bind to α2δ1 subunits of the voltage‐gated calcium channels of dorsal root ganglion (DRG) neurons. However, these compounds also came with reported adverse side effects.^13^ Approaches like *SCN9A* gene knockdown using antisense peptide nucleic acids have shown promising data in clinical trials but are only administered parenterally and have not yet reached the market. Consequently, there remains a pressing need for novel small molecules for neuropathic pain management and a high‐throughput screening method to efficiently identify drug candidates.

We have applied a sensory neuronal excitability screen using sensory neuron‐like cells generated from human induced pluripotent stem cells (iPSCs) derived from IEM patients. Mutations in the *SCN9A*, such as V400M and F1449V, increase sensory neuron excitability by altering the Na_V_ 1.7 channel's activation and inactivation voltage dependence, reducing the stimulus intensity required to induce nociceptive neuron firing, resulting in excessive spontaneous firing and chronic pain signalling, respectively. Modelling neuronal hyperexcitability in vitro using IEM patient‐derived iPSCs sensory neuron‐like cells harbouring one of these key mutations in *SCN9A* is likely to identify mechanisms useful beyond the small monogenic pain population, given the importance of neural excitability to pain signalling. Notably, our screening was conducted using patient‐derived iPSC sensory neurons from individuals with IEM, which were plated onto microelectrode arrays (MEAs). This approach, validated by other groups, has proven to be an effective platform for discovering IEM analgesics.^14^, ^15^, ^16^, ^17^, ^18^, ^19^, ^20^, ^21^ Screening of compounds from a chemogenomic small molecule library provided by AstraZeneca PLC identified four hit compounds that warrant further investigation as potential analgesics for IEM.

## 2 METHODS

### 2.1 Chemicals

All compounds applied to cell cultures in this study were dissolved in neuralisation medium (Table S1). AstraZeneca provided a curated set of 281 small molecules from their chemogenomic library, each shipped at a stock concentration of 10 mM in dimethyl sulfoxide (DMSO). The compound selection was specifically curated based on pharmacological activity targeting the human nervous system. For blinding purposes, each compound was anonymised and assigned a unique identifier consisting of the prefix ‘AZ’ followed by a three‐digit numerical code (e.g., AZ001, AZ002, AZ003; Table S1). This anonymisation ensured unbiased data acquisition and analysis. All chemicals were stored at −20°C and handled under sterile conditions.

### 2.2 iPSC culture

Human iPSCs were expanded on sterile, flat‐bottomed polystyrene TPP® tissue culture plates (Sigma–Aldrich; Z707759) coated with 20 µg/mL Matrigel® Matrix Basement Membrane Growth Factor Reduced (Corning; 354230) in DMEM/F12 (Gibco™; 11320033). Coating was performed according to the manufacturer's instructions.

The culture medium, mTeSR™1 (STEMCELL Technologies; 85850), was refreshed daily. iPSCs were maintained at 37°C with 5% CO₂ and 95% relative humidity. Cells were passaged upon reaching approximately 80% confluence using Versene™ EDTA (Gibco™; 15040066). Briefly, plates were washed once with DPBS without calcium and magnesium (Gibco™; 14190250) and incubated with Versene EDTA at 37°C for 3–5 min until colonies began to detach. Detached cells were gently resuspended, centrifuged at 200×*g* for 3 min, and reseeded at a ratio of 1:6 to 1:10, depending on colony morphology and density.

The control iPSCs used in this study, namely, WT1, WT2 and WT3, were reprogrammed from normal human dermal fibroblasts using non‐integrating Sendai virus and fully characterised as described previously.^22^, ^23^ IEM patient‐specific iPSC lines with *SCN9A* mutations, IEM1 (RCi001‐A; V400M mutation), IEM2 (RCi002‐A; F1449V mutation) and IEM3 (RCi002‐B; F1449V mutation), were obtained from the European Bank for Induced Pluripotent Stem Cells. Those were reprogrammed from human peripheral blood mononuclear cell‐derived erythroblasts using non‐integrating Sendai virus technology. IEM2 and IEM3 were derived from the same patient.

### 2.3 Neural induction of human iPSCs

Human iPSCs were rinsed with room temperature DPBS (Gibco™; 14190250) and dissociated to single cells using ACCUTASE™ (STEMCELL Technologies; 07920) for 5–10 min at 37°C. Cells were gently collected, centrifuged at 300×*g* for 5 min and resuspended in mTeSR™1 supplemented with 10 µM Y‐27632 (Chemdea; CD0141). Cells were seeded at a density of 100 000 cells/cm^2^ and cultured at 37°C with 5% CO₂. The medium was replaced daily until cells reached 90–100% confluence, marking day 0 of differentiation (Table S1). For the first 3 days of differentiation, cells were cultured in inhibition medium, supplemented with 100 µM 2‐mercaptoethanol (Gibco™; 31350‐010), 1 µM LDN193189 hydrochloride (Sigma–Aldrich; SML0559) and 10 µM SB431542 hydrate (Sigma–Aldrich; S4317). From day 3, the inhibition medium was further supplemented with 3 µM CHIR99021 (Sigma–Aldrich; SML1046), 10 µM DAPT (Sigma–Aldrich; D5942) and 10 µM SU5402 (Sigma–Aldrich; SML0443). On day 5, LDN193189 hydrochloride and SB431542 hydrate were no longer included in the daily medium replacement. From day 6, inhibition medium was slowly transitioned to neuralisation medium, 25% changes every 2 days and supplementation remained the same. On day 12, the cells were reseeded onto poly‐d‐lysine and laminin (PdL & L) (Sigma–Aldrich; 127‐2.5) coated surfaces at a density of 250 µL/cm^2^ in neuralisation medium supplemented with 25 ng/mL Recombinant human 𝛽‐nerve growth factor (𝛽‐NGF) (Peprotech; 450‐01), 25 ng/mL recombinant human/murine/rat brain‐derived neurotrophic factor (BDNF) (Peprotech; 450‐02), 25 ng/mL recombinant human neurotrophin‐3 (NT‐3) (Peprotech; 450‐03) and 25 ng/mL recombinant human glial‐derived neurotrophic factor (GDNF) (Peprotech; 450‐10), 100 µM 2‐mercaptoethanol and 3 µM CHIR99021. Fresh medium was replaced daily until day 14. On day 14, cells were exposed to 10 µg/mL mitomycin C from *Streptomyces caespitosus* (Sigma–Aldrich; M7949) for 2 h to inhibit cellular proliferation. After mitomycin C exposure, cells were rinsed with DPBS and transferred back to neuralisation medium. On day 16, supplementation with 25 ng/mL 𝛽‐NGF, BDNF, NT‐3, GDNF and 100 µM 2‐mercaptoethanol continued; however, CHIR99021 was omitted. Cells were maintained in neuralisation medium, refreshed twice weekly for a further 54 days, to facilitate functional neuronal maturation. On day 70, experimental endpoints (MEA, cytotoxicity, RNA‐Seq and patch clamp) were assessed.

### 2.4 Phase contrast and immunofluorescence microscopy

Phase contrast microscopy was performed using an Axiovert 200 inverted microscope (ZEISS). Phase contrast images were acquired under optimised illumination and magnification conditions suitable for observing live cell morphology and confluency.

For immunocytochemistry, cell cultures were gently rinsed with 4°C DPBS, followed by fixation with 4% (w/v) paraformaldehyde (Thermo Scientific™; 10342243) prepared in PBS for 10 min at room temperature. The fixed cultures were then washed three times for 5 min each with 4°C DPBS to remove residual fixative. To access intracellular antigens the cell membrane was permeabilised by the incubation of samples with 0.25% (v/v) Triton™ X‐100 (Sigma–Aldrich; X100‐100ML) in DPBS at room temperature for 30 min, followed by three 5‐min washes with DPBS.

To minimise non‐specific antibody binding, cultures were blocked with a 10% (v/v) solution of serum from the secondary antibody host species in DPBS for 60 min at room temperature. Primary antibodies were prepared in a 1% (v/v) solution of donkey serum in DPBS (Table S1) and applied to the samples, followed by overnight incubation at 4°C. After primary antibody incubation, cultures were washed and subsequently exposed to secondary antibodies, diluted in a 1% (v/v) donkey serum solution in DPBS for 60 min at room temperature while protected from light. Samples were then subjected to three additional 10‐min washes with DPBS in the dark to prevent photobleaching.

Nuclear counterstaining was performed using 1 µg/mL Hoechst 33342 (Thermo Scientific™; 62249) in DPBS for 15 min at room temperature, followed by a final DPBS wash. Samples were mounted on glass slides using VECTASHIELD^®^ Antifade Mounting Medium (Vector Laboratories; H‐1000‐10) to preserve florescence signals. Prepared slides were stored at 4°C, protected from light when not being examined.

Fluorescence images were acquired using an Axio Imager.Z1 fluorescence microscope (ZEISS) equipped with appropriate filter sets for antibody‐specific fluorophores. Image analysis and processing were performed using ZEISS Efficient Navigation (ZEN) software (ZEISS), ensuring optimal contrast, brightness and signal‐to‐noise ratio.

### 2.5 Cell viability

Cell viability was assessed using the CyQUANT™ lactate dehydrogenase (LDH) Cytotoxicity Assay Kit (Invitrogen; C20300) following compound exposure. After compound exposure, cells were rinsed with room temperature DPBS to remove residual compounds. Cells were then incubated in neuralisation medium (Table S1). Absorbance readings were taken at 490 nm (LDH activity) and 680 nm (background absorbance) using a spectrophotometer. Measurements were taken at 1, 6 and 24 h post‐exposure. LDH activity was calculated by subtracting background absorbance at 680 nm from absorbance at 490 nm. Data were normalised to vehicle controls and expressed as relative LDH activity. All steps were performed following the manufacturer's instructions to ensure consistency and reproducibility. A threshold LDH activity value of 5% of the LDH Positive Control included in the CyQUANT LDH Cytotoxicity Assay Kit was used to indicate the absence of toxicity which correlates with published neurotoxicity assays dependent on LDH release.^24^

### 2.6 MEA recordings

On day 12 of neural induction, sensory neuron‐like cells were dissociated and seeded onto PdL & L‐coated MEA plates (Multi Channel Systems; 24W700/100F‐288) at a density of 150 000 cells/cm^2^. Cells were fed twice weekly for 58 days. Recordings were conducted using the Multiwell‐MEA‐System and Multi‐Channel Experimenter software (Multi Channel Systems). All investigations taking place on the MEA were preceded by replacement of the cell culture medium on the day before the experiment. The MEA plates were incubated on the head stage of the system at 37°C and monitored for 30 min prior to compound exposure. Neuronal activity during the final 5 min of this equilibration period was recorded and served as a baseline. Compounds were then applied to the cultures, incubated at 37°C for 30 min before recording the activity for the final 5 min. All AZ compounds screened on the MEA platform were exposed to spontaneously active iPSC‐derived sensory neuron‐like cells generated from the IEM patient cell line RCi002‐A harbouring the F1449V heterozygous point mutation.

The Multiwell‐MEA system utilised 24‐well MEA plates, with each well containing 12 electrodes (for a total of 288 electrodes per plate). Voltage fluctuations detected by each electrode were digitised at 20 kHz and represented as individual channels, resulting in 12 channels per well. Channels were classified as active if their detected mean spiking frequency exceeded 0.33 Hz; those below this threshold were considered inactive.

To evaluate the effect of candidate compounds on neuronal activity, the screening was conducted at a standardised concentration of 10 µM to ensure consistency across compounds. For each experimental condition, recordings were conducted across a minimum of three and a maximum of five wells. The voltage files (µV) were filtered between 1 and 10 kHz and spikes were detected with a threshold crossing algorithm set to ±5 standard deviations (SDs) of the voltage baseline activity, with a dead time set to 1 ms pre‐trigger and 2 ms post‐trigger. The mean spike frequency was calculated for each channel before and after the compound application in recordings lasting 300 s each. Data analysis included: (i) the ratio of mean spiking frequencies after/before treatment (expressed as a percentage and shown as normalised activity in Table S1), (ii) the false discovery rate (FDR) corrected Wilcoxon *p* value between the list of frequencies before and after treatment, (iii) the *z* and robust *z*’‐scores and Cohen *d* values between these two‐time series (size effect evaluation) and (iv) the number of active channels. The selection of analgesic hits was performed using the indexes mentioned above using the following thresholds: normalised activity <50%, FDR corrected *p* < .01, robust *z*’‐score ← 1.96 and >1.96 (95% confidence interval), Cohen *d* > 0.80 (large effect) and number of active channels *n* ≥ 9. Compounds were categorised based on their effect size. A reduction in mean spiking frequency greater than 90% was classified as a high‐intensity hit, indicating near‐complete suppression of activity; reductions between 70 and 90% were classified as moderate‐intensity hits, reflecting substantial activity suppression; and reductions between 50 and 70% were designated as low‐intensity hits, corresponding to moderate inhibition.

Concentration–response relationships were modelled using a nonlinear regression algorithm implemented in MATLAB, which fitted compound concentration data against neuronal activity to minimise the sum of squared residual errors. The concentration ranges were as follows: high‐intensity range: 10, 8, 6, 4, 2, 0 µM; moderate‐intensity range: 12.5, 10, 7.5, 5, 2.5, 0 µM; low‐intensity range: 16.6, 13.3, 10, 6.6, 3.3, 0 µM (0 µM indicates DMSO vehicle control). The model estimated the Hill coefficient (*n*), reflecting the steepness of the dose–response curve, and the IC_50_, representing the concentration at which 50% inhibition was observed. These parameters were then applied to the Hill equation to generate dose–response curves, with raw data plotted on a logarithmic scale. The goodness‐of‐fit of each curve was evaluated using the *R* ^2^ value, where values approaching 1 indicated strong agreement between the model and observed data.

### 2.7 Patch‐clamp

On day 12 of neural induction, iPSC‐derived sensory neuron‐like cells were dissociated and plated onto PDL & L‐coated coverslips at a density of 150 000 cells/cm^2^ for maturation. Voltage and current recordings were performed at day 70 of differentiation using the whole‐cell configuration of patch‐clamp with standard bath and pipette solutions.

*Bath solution*: 144.8 mM sodium chloride (NaCl), 2.5 mM potassium chloride (KCl), 0.5 mM magnesium chloride (MgCl_2_), 1.2 mM calcium chloride (CaCl_2_), 5 mM d‐glucose and 10 mM 2‐[4‐(2‐hydroxyethyl)‐1‐piperazinyl] ethanesulphonic acid (HEPES), with a pH adjusted to 7.4 using 1 M sodium hydroxide.

*Pipette solution*: 140 mM KCl, 6 mM NaCl, 5 mM HEPES, 4 mM adenosine 5‐triphosphate disodium salt hydrate, 1.2 mM phosphocreatine disodium salt hydrate, 3 mM MgCl_2_, 1 mM CaCl_2_ and 5 mM ethylene‐glycol‐tetra acetic acid, with a pH adjusted to 7.2 with 1 M potassium hydroxide. All reagents were purchased from Fisher Scientific.

Electrophysiological studies were conducted in a continuously perfused experimental chamber at controlled room temperature 22 ± 0.5°C. Whole‐cell patch clamp recordings were performed using a Multiclamp 700B amplifier, Digidata 1400 A/D interface and pClamp 10.2 software. Signals were digitised at 10 kHz and low‐pass filtered at 2 Hz using an 8‐pole Bessel filter. Glass micropipettes were pulled from Harvard Apparatus glass capillaries with a 1.7 mm inner diameter using a Narishige PC‐10 heat puller (two‐step setting). Filled with pipette solution, the resistance ranged between 3 and 7 MΩ. Micropipettes were attached to a manual or automatic manipulator for precise cell targeting. Using the gap‐free protocol in the current‐clamp configuration with a holding current of 0 pA, recordings were taken for 60 s to assess for sAPs. Resting membrane potential (*V*_m_) was calculated as the mean voltage over a 30 s period. Neurons were classified as ‘quiet’ (no APs), ‘attempting sAP’ (classic AP shape with a sharp spike in *V*_m_ that recovers but does not reach 0 mV), or complete sAP (spikes that reached 0 mV).

A 1 s current step protocol was used to assess induced action potentials (iAPs). Neurons were initially hyperpolarised between −80 and −90 mV, then subjected to incremental current injections (−10 to 180 pA for iPSC‐derived sensory neuron‐like cells) with 30 ms pauses between steps. Neurons were classified based on their response as ‘quiet’, ‘attempting single iAP’, ‘complete single iAP’, ‘attempting train of iAP’ or ‘complete train of iAP’.^25^, ^26^ Spike parameters (overshoot, after‐hyperpolarisation, depolarising/repolarising rate, spike height, half‐height width and threshold) were analysed using Clampfit 10.2. The depolarising and repolarising rates were determined by differentiating the voltage with respect to time to find the maximum rising and declining slopes. The threshold was identified using the peak of the third derivative of voltage during the depolarising phase. The half‐height width was calculated at 50% of the spike height.

### 2.8 Recording of transmembrane currents using voltage‐step activation/inactivation protocol

Voltage‐gated sodium (Na_V_) and potassium (K_V_) currents were recorded using a standard voltage‐step activation/inactivation protocol in voltage‐clamp mode. Cells were held at −70 mV as the initial holding potential. The test potentials ranged from −120 to 80 mV, increasing in 5 mV increments per sweep (Figure S3A,B). To visualise the Na_V_ activation current, the membrane potential was stepped from −70 mV to the test potentials (−120 to +80 mV). The activation current was identified as a large inward spike at the first voltage step. The magnitude of the current was determined by the negative peak values. For Na_V_ inactivation currents, the current at the trough between the second and third held voltages was used; with the negative peak values indicating magnitude (Figure S3C). The K_V_ current was determined by taking the mean of the steady‐state currents during the last ∼50 ms of the held voltage step. The effects of tested AZ compounds shown in Figure 6E were normalised to the tetrodotoxin (TTX) effects (*I*/*I*_TTX_).

### 2.9 RNA extraction and bulk RNA‐Seq

Total RNA was extracted and purified using the ReliaPrep™ RNA Cell Miniprep System (Promega; Z6012) following the procedures outlined in the manufacturer's technical manual. Adherent cell cultures were washed with sterile 4°C DPBS to remove residual medium before lysis with BL buffer supplemented with 1‐thioglycerol. The resulting lysates were collected into individual microcentrifuge tubes and processed according to the ReliaPrep™ protocol, including DNase I treatment to eliminate genomic DNA contamination.

Purified total RNA was resuspended in nuclease‐free water, and its quality, purity and concentration were assessed using a NanoDrop™ 2000 Spectrophotometer (Thermo Scientific™; ND‐2000), a Qubit™ Flex Fluorometer (Invitrogen™; Q33327) and 2100 Bioanalyzer (Agilent; G2939BA). RNA integrity numbers greater than 8.0 were considered suitable for downstream library preparation.

RNA libraries were constructed using the NEBNext Single Cell/Low Input RNA Library Prep Kit for Illumina (New England BioLabs; E6420), according to the manufacturer's instructions. RNA samples were quantified and normalised to 10 ng total RNA using the Qubit™ Flex Fluorometer. Quality control of the resulting cDNA libraries was performed using a 4200 TapeStation (Agilent; G2991BA) to determine fragment size and integrity. Library molarity was calculated, and samples were equimolarly pooled into a single tube for bulk mRNA sequencing.

Sequencing was performed on an Illumina® NovaSeq™ 6000 system (20012850) using an S1 flow cell (Illumina®; 20028319), generating paired‐end reads (2 × 100 base pairs) with a sequencing depth of approximately 25 million single reads per sample.

Raw sequencing reads were pre‐processed, and raw counts were normalised using DESeq2 (Bioconductor) via the median of ratios method to ensure comparability of gene expression levels across samples. Log_2_ fold changes (LFC) greater than 2 were considered biologically significant for differential gene expression analysis between iPSCs and sensory neuron‐like cells. *p*‐values were adjusted for multiple comparisons with control the FDR.^27^

We compared bulk RNAseq data for the iPSC‐derived sensory neuron‐like cells with those of human DRG cells downloaded from GSE201586.^28^ Filtered matrix counts for each sample were pre‐processed using Seurat 5.2.1.^29^ Nuclei from each sample were retained if they had a total count > 1000, number of features (genes) > 1000 and if the percentage of mitochondrial reads <5%. Human DRG samples were then integrated using harmony 1.2.3.^30^ PCA and UMAP dimensionality reduction methods were applied to the integrated dataset, followed by the FindNeighbors and FindClusters function. All remaining human DRG samples clustered well together. The integrated object was then pseudobulked by using the AggregateExpression function from Seurat, using the RNA assay and grouped by sample name. The pseudobulk samples were then combined with our RNA‐Seq experiment using the bind_cols function from tidyverse 2.0.0^31^ and taking an intersection of the features present in the bulk RNA‐Seq and pseudobulk datasets. These counts were then imported into DESeq2 1.46.0^32^ using the DESeqDataSetFromMatrix function. Data were then transformed using the vst function followed by batch removal using limma 3.62.2^33^ with the removeBatchEffect function and batch = ‘dataset’. A PCA plot was then generated using the plotPCA function and visualised using ggplot2 3.5.1 (Figure S5).

### 2.10 Statistical analyses

Statistical analyses were performed using appropriate tests based on data distribution and experimental design. Two‐way ANOVA was used to compare means across groups with two independent variables, while Student's *t*‐tests analysed differences between two groups for normally distributed data. For non‐parametric data, the Wilcoxon signed rank test (paired comparisons), Mann–Whitney *U* test (independent groups) and Kruskal–Wallis test (multiple groups) were applied. Post hoc tests were conducted where necessary to identify specific group differences. All statistical tests and data analysis were performed using Excel (Microsoft Office 365), GraphPad Prism 10 (Dotmatics) and MATLAB (MathWorks; R2023a). Non‐linear regression analysis was performed to calculate half‐maximal inhibitory concentration (IC_50_) values and other dose–response relationships. Data are presented as mean ± standard error of the mean (SEM) or mean ± SD, as indicated in the figure legends. Statistical significance was assumed by a *p* value ≤ .05. Significance levels: ns = *p* > .05 (not significant), * = *p* ≤ .05, ** = *p* < .01, *** = *p* < .001 and **** = *p* < .0001. We calculated a robust standardised effect size (denoted robust *z*′) as the difference in medians between groups divided by the pooled median absolute deviation. This metric provides a nonparametric analogue to the classical *z*‐score and is suited for skewed or non‐normal distributions. It is distinct from the *z*‐prime (*z*′) statistic used in high‐throughput screening contexts. The number of replicates (*n*) corresponds to the number of experimental wells, unless otherwise indicated in the respective figure legends.

## 3 RESULTS

### 3.1 Generation of IEM‐specific and control sensory neuron‐like cells

**FIGURE 1:**
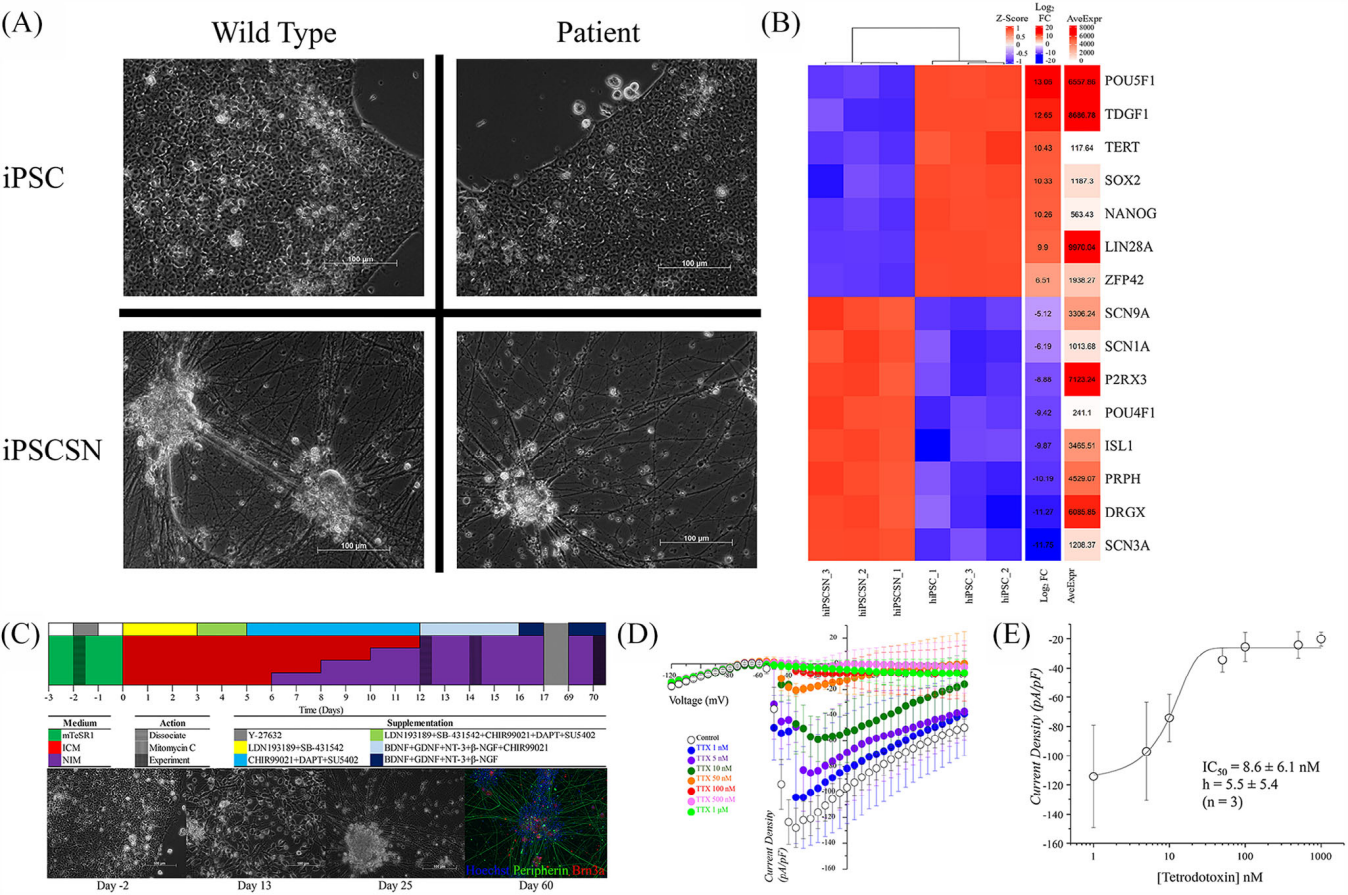
Morphological, transcriptomic and functional characterisation of wild‐type and IEM iPSC‐derived sensory neuron‐like cells. (A) Representative phase‐contrast images of iPSCs and iPSC‐derived sensory neuron‐like cells from wild‐type (WT3) and IEM‐iPSC lines (IEM2) are shown. The upper left panel displays WT3 iPSCs, while the lower left shows sensory neurons (iPSCSN) differentiated from WT3‐iPSCs. The upper right panel presents IEM2‐iPSCs and the lower right shows the iPSCSN differentiated from IEM2‐iPSCs. Morphological features, including neuronal soma size, neurite outgrowth and network formation, are illustrated. Images were acquired at 20× magnification, with scale bars = 100 µm. (B) Differential gene expression analysis: key differentially expressed genes (DEGs) indicative of sensory neuron phenotype were identified from RNA sequencing data, with up‐regulated genes with log₂ fold change (log FC) ≥ 2 shown in red, and down‐regulated genes with log FC ≤ −2 shown in blue. Average expression levels (AveExpr) across all samples are included to contextualise baseline expression. Samples were derived from the WT3 cell lineage, with labels indicating ‘hiPSCSN_’ for sensory neuron‐like cells and ‘hiPSC_’ for undifferentiated cells (*n* ≥ 3, *p* ≤ .0001). (C) Differentiation protocol for sensory neuron‐like cells: a modified differentiation protocol was used to generate excitable sensory neuron‐like cells from iPSCs. Immunofluorescence staining confirmed the presence of key neuronal markers: Peripherin (green), Brn3a (red) and Hoechst (blue) as a nuclear counterstain. ICM: inhibition medium. NIM: neuralisation medium. Media compositions shown in Table S1. (D) Current density–voltage characteristics of Na^+^ current: voltage‐gated sodium (Na^+^) currents were recorded in human IEM1 iPSC‐derived sensory neuron‐like cells using whole‐cell patch‐clamp electrophysiology. The current density–voltage (*I*–*V*) relationship was assessed under varying concentrations of tetrodotoxin (TTX) to evaluate TTX‐sensitive Na^+^ channels. (E) Concentration–response characteristics for TTX inhibition: Boltzmann function fitting was applied to model the TTX concentration–response relationship for peak Na^+^ current density in human IEM1‐iPSC‐derived sensory‐like neurons. Data are presented as mean ± SEM, with *n* ≥ 3 replicates per condition. Detailed experimental parameters and statistical analyses are provided in the methods section.

**FIGURE 2:**
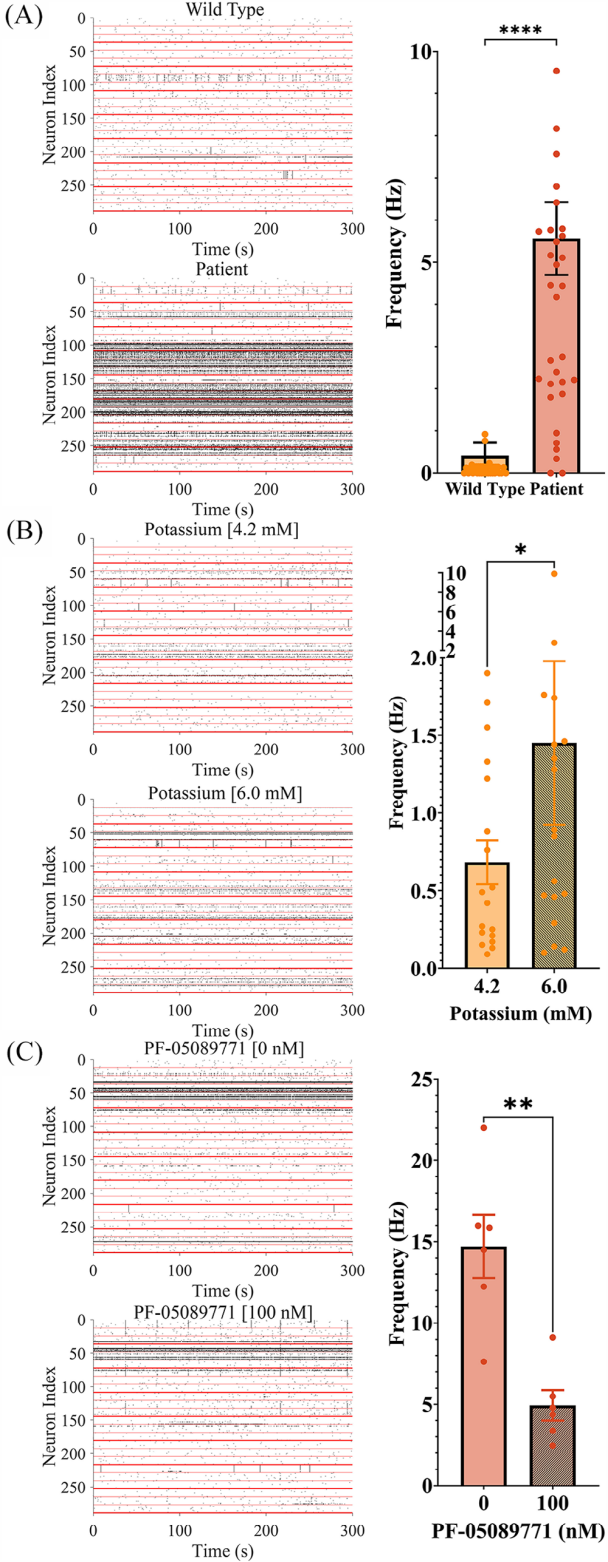
Hyperexcitability and pharmacological modulation of iPSC‐derived sensory neuron‐like cells. (A) Spontaneous activity differences between wild‐type and IEM patient‐derived sensory neuron‐like cells: spontaneous neuronal activity was recorded using a MEA system to compare activity patterns between wild‐type (WT) and IEM iPSC‐derived sensory neuron‐like cells. Upper left panel shows a raster plot of spontaneous firing activity in WT3 iPSC‐derived sensory neuron‐like cells over a 5‐min recording period. The lower left panel depicts a raster plot indicating increased spontaneous firing activity in IEM2 patient iPSC‐derived sensory neuron‐like cells under identical recording conditions. The histogram quantifies the frequency of firing across MEA wells in WT3 and IEM2 sensory neuron‐like cells, highlighting significant hyperexcitability in patient‐derived cells. Data are presented as mean ± SEM, with *N* ≥ 3 wells per condition, *n* ≥ 9 active channels, *p* ≤ .0001. (B) Manipulation of the electrochemical gradient: neuronal excitability was modulated by altering the extracellular potassium (K^+^) concentration to evaluate sensitivity to electrochemical gradients. The upper raster plot shows neuronal firing patterns of WT3 iPSC‐derived sensory neuron‐like cells in the presence of 4.2 mM KCl. The lower raster demonstrates increased neuronal firing activity in response to an elevated 6.0 mM KCl concentration. The histogram compares firing frequencies across different KCl concentrations, demonstrating a dose‐dependent increase in neuronal excitability. Data are presented as mean ± SEM, with *N* ≥ 3 wells per condition, *n* ≥ 9 active channels, *p* ≤ .05. (C) Inhibition by PF‐05089771: The effect of PF‐05089771, a potent and selective Na_V_ 1.7 sodium channel blocker, on neuronal hyperexcitability was assessed in IEM2 and IEM3‐iPSC‐derived sensory neuron‐like cells, the responses were pooled. IEM2 and IEM3 were prioritised for screening as these generated the most spontaneously active sensory neurons. The upper raster plot shows baseline spontaneous activity before PF‐05089771 (100 nM) exposure and the lower raster depicts reduced spontaneous activity following exposure to 100 nM PF‐05089771. The histogram shows a significant reduction in spike frequency after exposure to PF‐05089771. Data are presented as mean ± SEM, with *N* ≥ 3 wells per condition, *n* ≥ 9 active channels, *p* ≤ .01. The Wilcoxon signed‐rank test was used for paired experiments, while the Mann–Whitney *U* test was applied for unpaired comparisons of ranks.

To establish a reliable experimental model of human nociceptive neurons, sensory neuron‐like cells were derived from iPSCs obtained from two IEM patients carrying *SCN9A* mutations and three control unaffected subjects (Figure 1A). The pluripotent status of these iPSCs was confirmed by the expression of pluripotency markers *POU5F1 (OCT‐4)*, *TDGF1*, *TERT*, *SOX2*, *NANOG*, *LIN28A* and *ZFP42*, as shown by bulk RNA‐Seq analysis (Figure 1B). All iPSCs were differentiated into neurons with phenotypes resembling DRG nociceptive neurons using a protocol that draws insights from several previously published studies, supplemented with small molecules, growth factors and 2‐mercaptoethanol at specific time points (Figure 1C).^10^, ^34^, ^35^, ^36^, ^37^ Within 13 days, this method generated cells with neuronal morphology (Figures 1A and S1A), which were mitotically inactivated with 10 µM Mitomycin C and matured until day 70 of differentiation. By this time, the cells were capable of generating sAPs, as measured using the MEA platform (Figures 2 and S1B,C). Treatment with the glycogen synthase kinase 3 inhibitor, CHIR99021, for 13 days resulted in a higher density of neuronal projections compared with 9 days of treatment (Figure S1A). MEA recordings demonstrated that denser projections led to significantly more active neurons without altering the spiking rate (Figure S1B,C). Thus, the 13‐day treatment was used for the remainder of the study. Immunohistochemical analysis supported a sensory neuronal phenotype in the differentiated cells, based on Islet 1, Brn3a and Peripherin expression (Figures 1C and S2). Transcriptomic analysis by bulk RNA‐Seq revealed the expression of key DRG markers such as DRG homeobox protein (*DRGX*), *SCN3A*, *P2RX3* and the Na_V_ 1.7 channel‐encoding *SCN9A* (Figure 1B).^38^ Transcriptomic comparison demonstrated a high degree of similarity between the iPSC‐derived sensory neuron‐like cells and human *DRG* neurons (Figure S5).

### 3.2 Characterisation of IEM iPSC‐derived sensory neuron‐like cells using patch‐clamp electrophysiology

To explore the functional characteristics of IEM iPSC‐derived sensory neuron‐like cells, whole‐cell patch‐clamp electrophysiological experiments were performed. Using a gap‐free current‐clamp (*I* = 0 pA) protocol we recorded the resting membrane potential (*V*_m_) and ongoing electrical activity. This analysis revealed a resting membrane potential of IEM iPSC‐derived sensory neuron‐like cells close to that of the DRG sensory neurons. IEM iPSC‐derived sensory neuron‐like cells were categorised based on their activity levels: 67% (29 out of 43) were ‘quiet’ showing no activity; 7% (three out of 43) were ‘attempting’, exhibiting only sub‐threshold transient depolarising events; and 26% (11 out of 43) were ‘spontaneous’, displaying overshooting action potentials (Table S2). Under an induced activity protocol, no IEM iPSC‐derived sensory neuron‐like cells remained quiet. 5% (two out of 42) were classified as ‘Attempting Singles’, 50% (21 out of 42) as ‘Single iAP’, 2% (one out of 42) as ‘Attempting Trains’ and 43% (18 out of 42) as ‘Trains’ (Table S3). The passive and active parameters of IEM iPSC derived sensory neuron‐like cells confirmed their genuine neuronal properties (Table S4).

To explore the inhibitory effects of AZ‐compounds on Na_V_ current in IEM iPSC‐derived sensory neuron‐like cells the voltage‐step protocol, with the holding potential −70 mV and repetitive 10 ms‐long steps to −35 mV to achieve the maximal Nav current amplitude, was employed. The involvement of the Na_V_ 1.7 channel was further supported by the application of TTX, an established Nav channel antagonist, which caused a significant reduction in the firing rate (Figure 1D,E).

### 3.3 Electrophysiological characterisation of IEM‐specific and control sensory neuron‐like cells using MEA

Cultured on MEAs, human IEM iPSC‐derived sensory neuron‐like cells enabled compound screening for potential analgesics. IEM sensory neuron‐like cells showed a higher proportion of active channels in MEA recordings compared with wild‐type controls (Figure 2A). Manipulating the electrochemical gradient with extracellular KCl increased the spike frequency of control iPSC‐derived sensory neuron‐like cells (Figure 2B), while the Na_V_ 1.7 channel blocker PF‐05089771 (100 nM) significantly reduced the firing rate of IEM iPSC‐derived sensory neuron‐like cells and a decreased proportion of active channels in each well (Figure 2C). IEM iPSC‐derived sensory neuron‐like cells exhibited a proportional relationship between temperature and firing rate (Figure S4A,B), consistent with IEM patients’ pain perception, whereas control iPSC‐derived sensory neuron‐like cells showed lower increases in firing rate with temperature (Figure S4A,C). This characterisation highlighted the utility of a MEA‐based model for screening small molecule libraries to identify candidates capable of decreasing the firing rate of IEM‐specific iPSC‐derived sensory neuron‐like cells.

### 3.4 Partial screening of a pharmacogenetic small compound library

**FIGURE 3:**
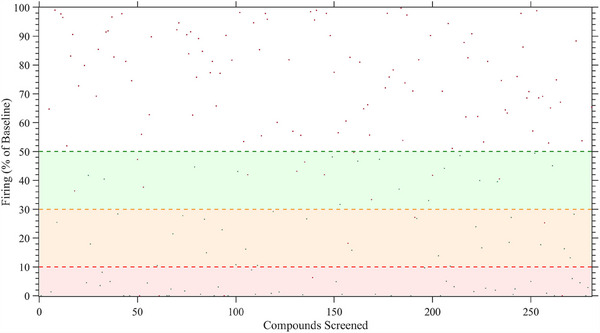
Screening and identification of effective inhibitors of spontaneous activity in IEM2 iPSC‐derived sensory neuron‐like cells. Compound screening: A total of 281 compounds were screened for their effects on spontaneous firing activity in IEM2 iPSC‐derived sensory neuron‐like cells. Each compound was tested at a concentration of 10 µM. Neuronal activity was assessed by calculating the mean frequency of voltage spikes recorded across active electrodes and determining the proportion of activated electrodes within the neuronal network. Activity measurements were obtained from the final 5 min of a 30‐min incubation period to ensure steady‐state effects were captured. The dashed green line represents a 50% reduction threshold in spontaneous activity, while the orange line shows greater than 70% inhibition, and the red dashed line denotes the threshold for greater than 90% inhibition of baseline activity. Compounds falling into these categories are further highlighted by green, orange or red shading of the areas below the respective dotted lines. Compounds were classified as effective inhibitors based not only on their inhibition potency but also on meeting statistical criteria: FDR‐adjusted *p* value ≤ .01, a robust *z*′ ≤ 1.96, a Cohen's *d* score ≥ 0.80, and a median separation ≥2× median absolute deviation (MAD). Those deemed worthy hits are highlighted as green dots, while compounds that did not meet the criteria are marked as red dots. All recordings were taken from *N* ≥ 3 wells per condition, *n* ≥ 9 active channels.

**FIGURE 4:**
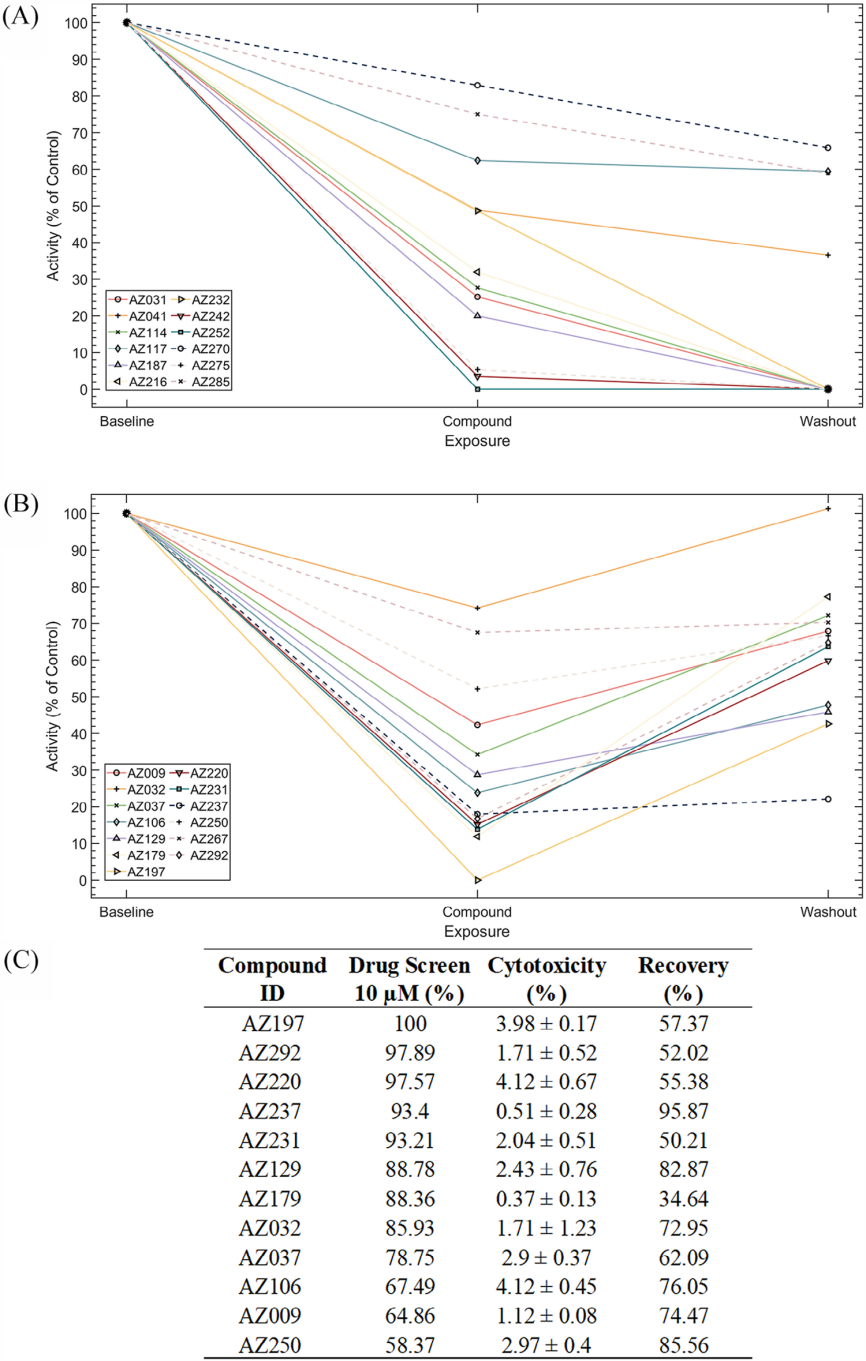
Reversibility of inhibition in iPSC‐derived sensory neuron‐like cells following exposure to identified compounds. The reversibility of inhibition was assessed by measuring the recovery of spontaneous spike activity in day 70 iPSC‐derived sensory neuron‐like cells after a 24‐h washout period following 10 µM compound exposure. Spontaneous spike activity was recorded and analysed to determine whether inhibitory effects persisted or were reversed after compound removal. Irreversible inhibitors: Compounds categorised as irreversible inhibitors failed to recover spiking activity after the 24‐h washout period, indicating sustained inhibitory effects even in the absence of continued compound exposure. These compounds are characterised by prolonged binding or irreversible interaction with their molecular targets. Reversible inhibitors: Compounds classified by either partial or complete return to baseline spike activity after the 24‐h washout period, suggesting transient and reversible molecular interactions with their targets. A total of 12 compounds showing significant and reproducible effects on spike activity, with varying degrees of reversibility, were identified as candidates for further investigation to elucidate their inhibitory mechanisms and pharmacological properties. Drug screen 10 µM shows the percentage change in neuronal activity caused by the compound at a concentration of 10 µM, compared with the baseline activity. This value indicates the inhibitory effect of the compound. Cytotoxicity indicates the percentage of cell death or damage observed at 10 µM, representing the compound's toxic effects on the cells. Recovery provides the percentage of neuronal activity that returned to baseline after removing the compound, indicating the reversibility of the compound's effects. (A–C) Data are presented as mean values, derived from day 70 iPSC‐derived sensory neuron‐like cells (IEM2) (mutation F1449V) with *n* ≥ 3 replicates per compound.

We screened 281 compounds on sensory neuron‐like cells derived from an IEM patient (RCi002‐A) carrying the F1449V mutation in the *SCN9A* gene. While we were blinded to the identities of the compounds, they were provided by AstraZeneca from their curated chemogenomic library, specifically designed to target genes, proteins or pathways. In our case, the selected compounds targeted pharmacological pathways in the human nervous system based on previously reported or suspected neuronal impact. Compounds reducing spike activity by more than 50% of maximal firing over 5 min after 30 min of exposure with statistical significance (*p* value < .01), robust *z*’‐score of <−1.96 or >1.96, and Cohen *d* > 0.8 were considered effective, in alignment with common practices in toxicological screening. Initial screening identified 85 inhibitors, with 49% showing intense inhibition (90–100%), 32% showing moderate inhibition (90–70%) and 19% reducing spontaneous firing by 70–50% (Figure 3). Cytotoxicity assessments based on LDH secretion narrowed the candidate pool to 25 compounds (Table S1). Of these, 12 compounds caused irreversible inhibition, while 13 allowed functional recovery after a 24‐h washout period, indicating at least partially reversible inhibition (Figure 4A,B). Reversibility was defined as an increase of neuronal activity following washout and compounds showing this were selected in preference to those that showed no increase in neuronal activity following washout. We prioritised these reversible compounds to exclude those that, while non‐cytotoxic, appeared to irreversibly bind to their targets, induce receptor desensitisation, exert prolonged downstream signalling effects or cause broad non‐specific cellular modulation.

**FIGURE 5:**
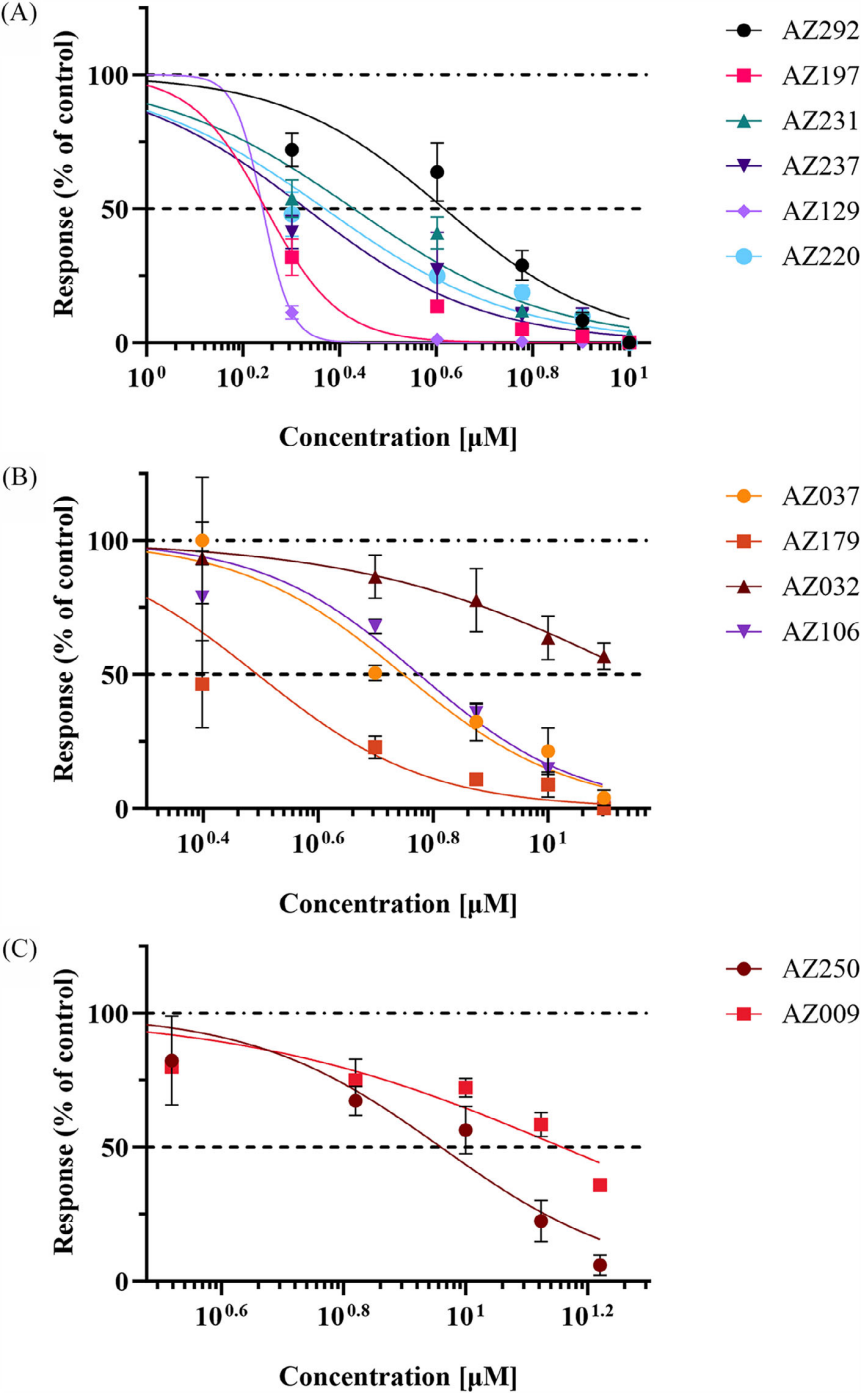
Concentration–response relationships for selected compounds in IEM2‐derived sensory neuron‐like cells. Non‐linear regression modelling was performed using the Boltzmann equation to analyse the effects of increasing compound concentrations on spontaneous spike generation in IEM iPSC‐derived sensory neuron‐like cells harbouring the F1449V mutation in *SCN9A*. Spike frequency was recorded following compound exposure, and responses were normalised to baseline activity for comparison across compounds. (A) High‐intensity inhibitors: Compounds AZ292, AZ197, AZ231, AZ237, AZ129 and AZ220 demonstrated pronounced inhibitory effects on spontaneous spike generation, with clear dose‐dependent responses and lower IC_50_ values, indicating high potency. (B) Moderate‐intensity inhibitors: Compounds AZ037, AZ170, AZ032 and AZ106 exhibited moderate inhibitory effects, characterised by dose–response curves with intermediate IC_50_ values. (C) Low‐intensity inhibitors: Compounds AZ250 and AZ009 displayed higher IC_50_ values when analysing the effect on spontaneous spiking activity compared with the other compounds. Data are presented as mean ± SEM, with *n* ≥ 3 replicates per compound.

Concentration–response curves for the 13 reversible compounds were generated, though AZ267 did not show a concentration‐effect relationship, leaving 12 compounds for further analysis (Figure 4C). Validation using the Na_V_ 1.7 inhibitor, PF‐05089771, showed a half maximal inhibitory concentration (IC_50_) of 80.1 nM, consistent with literature values (data not shown).^10^, ^39^ IC_50_s, inhibitory concentration ranges, *R* ^2^ coefficients and *p* values for selected compounds are shown in Table S1, with concentration–response curves shown in Figure 5A–C. Compounds with IC_50_s ≤ 8 and functional recovery ≥60% were selected for further patch‐clamp analysis, resulting in four candidates AZ129, AZ237, AZ106 and AZ037 (Table S1) for further assessment by patch‐clamp analysis.

### 3.5 Patch‐clamp analysis of the AZ‐compounds inhibition of Na_v_ currents in IEM iPSC‐derived sensory neuron‐like cells

**FIGURE 6:**
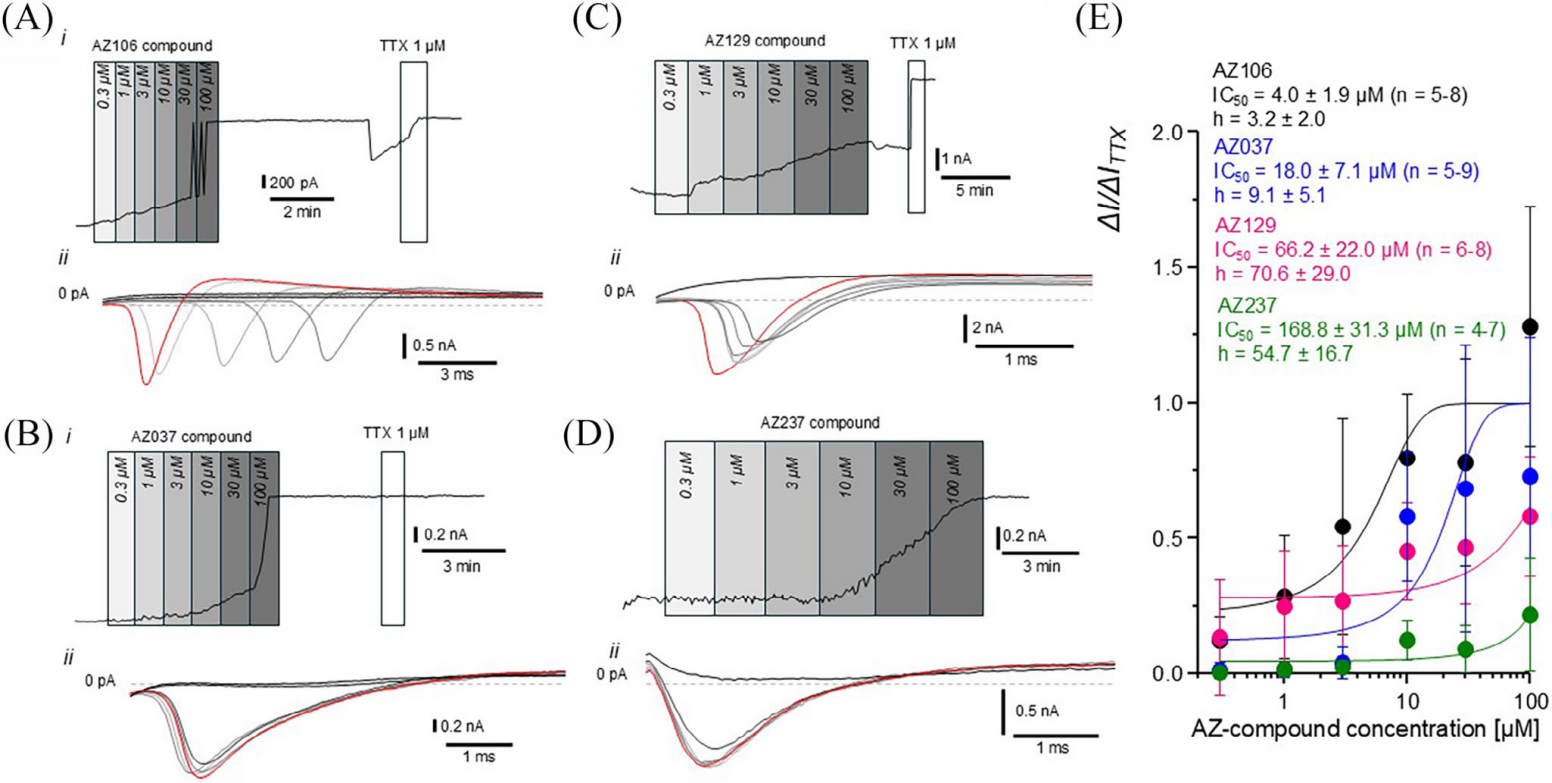
Effects of AZ106, AZ129, AZ037 and AZ237 on maximal voltage‐gated Na^+^ current in IEM iPSC‐derived sensory neuron‐like cells. (A) (i) Concentration‐dependent time course of AZ106‐compound inhibition of maximal voltage‐gated Na^+^ current at −35 mV in IEM iPSC‐derived sensory neuron‐like cells verified with inhibition by TTX 1 µM; (ii) concentration‐dependent inhibition of peak Na^+^ current by AZ106 compound at depolarising step from −70 to −35 mV in IEM iPSC‐derived sensory neuron like cells verified with inhibition by tetrodotoxin (TTX) 1 µM. Red trace represents the control voltage‐gated Na^+^ current, the current traces in the presence of AZ106 compound are shown from light to dark grey, the current in the presence of TTX 1 µM is black. (B) (i) Concentration‐dependent time course of AZ129 compound inhibition of maximal voltage‐gated Na^+^ current at −35 mV in IEM iPSC‐derived sensory neuron‐like cells verified with inhibition by TTX 1 µM; (ii) Concentration‐dependent inhibition of peak Na^+^ current by AZ129 compound at depolarising step from −70 to −35 mV in IEM iPSC‐derived sensory neuron like cells verified with inhibition by tetrodotoxin (TTX) 1 µM. Red trace represents the control voltage‐gated Na^+^ current, the current traces in the presence of AZ129 compound are shown from light to dark grey, the current in the presence of TTX 1 µM is black. (C) (i) Concentration‐dependent time course of AZ037 compound inhibition of maximal voltage‐gated Na^+^ current at −35 mV in IEM iPSC‐derived sensory neuron‐like cells verified with inhibition by TTX 1 µM; (ii) Concentration‐dependent inhibition of peak Na^+^ current by AZ037 compound at depolarising step from −70 to −35 mV in IEM iPSC‐derived sensory neuron like cells verified with inhibition by TTX 1 µM. Red trace represents the control voltage‐gated Na^+^ current, the current traces in the presence of AZ037 compound are shown from light to dark grey, the current in the presence of TTX 1 µM is black. (D) (i) Concentration‐dependent time course of AZ237 compound inhibition of maximal voltage‐gated Na^+^ current at −35 mV in IEM iPSC‐derived sensory neuron‐like cells; (ii) Concentration‐dependent inhibition of peak Na^+^ current by AZ237 compound at depolarising step from −70 to −35 mV in IEM iPSC‐derived sensory neuron‐like cells. Red trace represents the control voltage‐gated Na^+^ current, the current traces in the presence of AZ237 compound are shown from light to dark grey. (E) Concentration–response summary curve of AZ106, AZ037, AZ129 and AZ237 compounds on maximal voltage‐gated Na^+^ current at −35 mV in IEM iPSC‐derived sensory neuron‐like cells normalised to the inhibition caused by TTX 1 µM (Δ*I*/Δ*I*_TTX_). AZ106 compound is represented with black circles and black Boltzmann fitted line IC_50_ = 4.0 ± 1.9 µM (*n* = 5–8), *h* = 3.2 ± 2.0; AZ037 compound is represented with blue circles and blue Boltzmann fitted line IC_50_ = 18.0 ± 7.1 µM (*n* = 5–9), *h* = 9.1 ± 5.1; AZ129 compound is represented with purple circles and purple Boltzmann fitted line IC_50_ = 66.2 ± 22.0 µM (*n* = 6–8), *h* = 70.6 ± 29.0; AZ237 compound is represented with green circles and green Boltzmann fitted curve, 168.8 ± 31.3 µM (*n* = 4–7), *h* = 54.7 ± 16.7.

Patch‐clamp analysis of the four selected compounds revealed that AZ106, targeting the Na_V_ 1.7 channel, had the greatest effect with an IC_50_ of 4.0 ± 1.9 µM and a slope factor of 3.2 ± 2.0 (*n* = 5–8) (Figure 6A,E). AZ037 had the second greatest effect with an IC_50_ of 18.0 ± 7.1 µM and a slope factor of 9.1 ± 5.1 (*n* = 5–9) (Figure 6C,E), though it did not fully inhibit Na^+^ flow, as indicated by *I*/*I*_TTX _= 1.0. AZ129 had the third highest response with an IC_50_ of 66.2 ± 22.0 µM and a slope factor of 70.6 ± 29.0 (*n* = 6–8) (Figure 6B,E), followed by AZ237 with an IC_50_ of 168.8 ± 31.3 µM and a slope factor of 54.7 ± 16.7 (*n* = 4–7) (Figure 6D,E). The discrepancy between the IC50 values calculated for patch clamp analysis and MEA analysis for AZ129 and AZ237 are notable. This could be explained by the diverse nature of the recorded signals and rather different experimental conditions for MEA and patch‐clamp. MEA was used as a preliminary technique to point out the most effective compounds that attenuate the frequency of spontaneous firing of the hiPSC‐derived sensory‐like neurons. MEA recordings were performed at a physiological‐like temperature (∼37°C). Whereas patch‐clamp was selected as the most adequate approach to examine the effectiveness of AZ‐compounds specifically on the Na_v_ 1.7 currents in the whole‐cell configuration of voltage‐clamp mode. Patch‐clamp experiments were conducted at controlled room temperature (∼22°C).

## 4 DISCUSSION

### 4.1 Comprehensive characterisation of sensory neuron‐like cells

This study achieved a thorough morphological, molecular and functional characterisation of iPSC‐derived sensory neuron‐like cells. High gene expression of *SCN9A* and *SCN10A*, functional inhibition and activation of the Na_V_ 1.7 sodium ion channel, and consistent morphological features affirm the model's capacity to generate iPSC‐derived sensory neuron‐like cells. The phenotypic differences observed between wild‐type controls and IEM patient‐derived sensory neuron‐like cells are consistent with the project hypothesis and clinical reports, reinforcing the validity of the model. Differential gene expression analysis, highlighting down‐regulation of genes linked to pluripotency and up‐regulation of genes typical of sensory neurons in the DRG such as such as *DRGX*, *ISL1*, *POU4F1 (BRN3A)*,^40^ *SCN9A*, *LIX1* ^41^ and *P2RX3*,^42^ underscores the successful implementation of the differentiation protocol. The model's reliability is further supported by its consistency with literature and anticipated phenotypic outcomes.^14^, ^15^, ^16^, ^17^, ^18^, ^19^, ^20^, ^21^ Furthermore, comparison of RNA‐Seq data from IEM patient‐derived sensory neuron‐like cells with published RNA‐Seq data sets from human DRG neurons underlines the similarity between these two groups of cells.

### 4.2 Preliminary toxicity assessment of hit compounds

The high attrition rate in clinical trials, often due to toxicity, necessitates thorough preclinical assessments. We conducted a preliminary toxicity evaluation of hit compounds by measuring LDH release from iPSC‐derived sensory neuron‐like cells and assessing their ability to recover spiking activity after compound removal. Although these assessments do not provide a comprehensive toxicity profile (e.g., hepatotoxicity or cardiotoxicity), they offer valuable insights into the compounds’ neurotoxicity, particularly towards DRG neurons. Compounds that induce significant toxic responses or inhibit neuron activity recovery were deemed unsuitable as analgesics and excluded from further consideration. A potential shortcoming of our approach was that toxicity assessment was performed at the same compound concentration as MEA screening (10 µM) since this could preclude compounds which may not have been toxic at their effective concentrations; however, since our primary screen was to identify compounds capable of reducing spontaneous activity of IEM patient‐derived sensory neuron‐like cells, we chose to use a single concentration for toxicity assessment.

### 4.3 Validation of MEA screening approach

To validate the MEA screening approach, we measured the impact of hit compounds on the firing rate of iPSC‐derived IEM sensory neuron‐like cells using patch‐clamp quantification of membrane potential changes. AZ106 emerged as the most effective, reducing spiking activity with a potency approximately 100 times greater than lidocaine, which has an IC_50_ of 500 µM under similar conditions ^43^. Patch clamping also confirmed the reversibility of AZ106's action, similar to AZ129. While the precise mechanism of AZ106 remains unconfirmed, personal communication with AstraZeneca suggests it acts as a non‐specific Nav channel inhibitor, targeting proteins synthesised by *SCN1‐10A* and *SCN1‐10B*, including *SCN9A* which encodes the Na_V_ 1.7 channel, implicated in erythromelalgia. It is of course possible that AZ106 could bind to and inhibit the activities of other VGSCs such as Na_V_ 1.6 and Na_V_ 1.8 which are also expressed in sensory neurons with a phenotype similar to the DRG. Na_V_ 1.6 contributes to the TTX‐sensitive sodium current but is expressed at a lower level than Na_V_ 1.7 in the DRG^44^ so inhibition of this VGSC is less likely to induce the significant reduction in spike activity we observed in this study. Data provided by Astra‐Zeneca suggest that AZ106 may also bind Na_V_ 1.8 and from the results obtained in our study, we cannot rule out a potential contribution from the TTX‐resistant Na_V_ 1.8 channel; however, the significant reduction in spike frequency observed after exposure to the Na_V_ 1.7 specific inhibitor PF‐05089771 implies a strong (although not exclusive) contribution from Na_V_ 1.7 in the iPSC‐derived IEM sensory neuron‐like cells. Moreover, the Na_V_ 1.8 encoding gene (*SNC10A*) has been reported to be expressed at lower levels than *SCN9A* in DRG neurons.^45^ Furthermore, according to our experiments on the basic electrophysiological properties of the iPSC‐derived sensory neuron‐like cells, the dominant transmembrane conductances were represented with outward current through the voltage‐gated potassium channels and inward current through the VGSCs. The dominant subtype of the subunits assembling VGSCs was Na_v_ 1.7. AZ‐compounds had no significant effects on outward voltage‐gated potassium transmembrane current but displayed quite selective affinity to the inward voltage‐gated sodium current of the iPSC‐derived sensory neuron‐like cells. Therefore, considering these data, we have concluded that the tested AZ‐compounds selectively inhibited Na_v_ 1.7. The impact of AZ‐compounds on the function of other ion channels was not investigated, hence we cannot confirm that the MEA approach can be used to screen for targets other than Na_V_ 1.7 without further studies. While our manuscript was under review, a new study has demonstrated that human iPSC‐derived sensory neurons can provide a powerful platform for high‐throughput analgesic drug screening using the MEA platform. Similarly to us, the authors of this study show response to analgesic targets of ion channels (Na_V_, Ca_V_, K_v_ and transient receptor potential cation channel subfamily vanilloid type 1 (TRPV1).^15^ In this study, we ruled out compounds whose effects were irreversible up to 24 h after a 30‐min exposure to iPSC‐derived sensory neuron‐like cells. A caveat of this approach is that some of the compound may not have had sufficient time to elicit an irreversible effect within 30 min; however, as we aimed to identify rapidly acting compounds (after all, pain killers should be effective over short timescales), we chose 30 min exposure even if this meant not identifying potentially interesting compounds.

### 4.4 Receptor binding profiles and pain mechanisms

AstraZeneca's reports indicate that AZ106 binds to multiple receptors, including histamine receptor type 3 (H3R), acetylcholinesterase, muscarinic cholinergic receptors (1–3 & 5), sigma non‐opioid intracellular receptor 1, melanin concentrating hormone receptor 1, somatostatin receptor 4, dopamine receptor (D3R), hydroxytryptamine (5‐HT) receptors (1B, 2A and 4) and adrenoreceptor alpha 2A (ADRA2A). Several of these receptors are involved in pain mechanisms. For instance, somatostatin 4 receptor (SST4) has been shown to modulate TRPV1 currents in DRG neurons,^46^ and systemic administration of muscarinic receptor ligands induces analgesia.^47^ SST4 is expressed in the peripheral and central nervous systems, inhibiting sensory neuron activation without contributing to the endocrine roles of the somatostatin neuropeptide, making SST4 agonists appealing for pain modulation.^48^, ^49^, ^50^ Similarly, 5‐HT receptors produce anti‐nociceptive effects in the spinal column.^51^ While some receptors such as ADRA2A and D3R are involved descending neuronal pathways, sigma non‐opioid intracellular receptor 1 seems to be involved in regulating the interaction between nociceptive neurons and non‐neuronal inflammatory cells.^46^, ^47^, ^52^, ^53^ Activation of H3Rs on the spinal terminals of Aβ fibres in the DRG reduces nociceptive response to low intensity mechanical stimuli and inflammatory stimuli^54^; so it is conceivable that they may affect spiking in our model, and further investigation is required to understand the binding and downstream effects of AZ106 on these receptors.

An alternative possibility is that AZ106, AZ129, AZ037 and AZ237 create perturbations in the lipid bilayer membrane of IEM iPSC‐derived sensory neuron‐like cells. Several amphiphilic plant phenols are reported to inhibit the activity of VGSCs by integrating into the membrane and weakening bilayer integrity;^55^ however, further work to demonstrate the ability of these hits compounds to disrupt membrane structure would be needed to support this possibility.

### 4.5 Comparative analysis of binding targets

AZ106 shares several binding targets with AZ129, such as sigma non‐opioid intracellular receptor 1, potassium voltage‐gated channel subfamily H member 2 (KCNH2), SST4, D3R, muscarinic cholinergic receptors (1, 2 and 5), 5‐HT receptor 4 and ADRA2A. However, AZ129 also binds to additional 5‐HT receptors (1B, 1D, 2B, 2C, 3A, 7) and histamine receptor types 1 and 2 (H1R, H2R) and cannabinoid receptor 1 and opioid receptors κ1 and μ1 (all alternative targets provided by personal communication from Astra‐Zeneca PLC), which are all expressed in DRG neurons.^56^, ^57^ Despite shared targets, AZ129 has a lower IC_50_ than AZ106 and cannot completely inhibit VGSC within the range of concentrations tested. This suggests AZ129's receptor binding may be less effective in reducing action potential generation, supporting the putative role of AZ106 as a VGSC inhibitor. It is noteworthy that all the compounds identified in this study have IC_50_ values that are considerably higher than those of the Na_V_ 1.7 specific inhibitor PF‐05089771. This might imply that AZ106, AZ129, AZ037 and AZ237 would be less efficacious pain‐modulating drugs than PF‐05089771; however, reports of the analgesic activity of PF‐05089771 are equivocal^12^, ^58^ suggesting that the lower IC_50_ of PF‐05089771 may not be an absolute indicator of its efficacy.

### 4.6 Further investigation of additional compounds

AZ037 binds to KCNH2, opioid receptor δ1, opioid receptor μ1 and muscarinic cholinergic receptor 2. However, its sodium channel current data are highly variable, and its IC_50_ is lower than AZ106, complicating the interpretation of its binding activities. AZ237, with the lowest IC_50_ in the cohort that was assessed by patch clamping, binds to the Na_V_ 1.8 channel, crucial for action potential maintenance in DRG neurons, and solute carrier family 6 proteins (1, 11 and 13), responsible for γ‐aminobutyric acid (GABA) transport.^59^, ^60^, ^61^, ^62^, ^63^, ^64^ Infusion of GABA into the DRG of rats has been shown to inhibit nociceptive transmission^44^, ^63^ and DRG sensory neurons can synthesise, release and respond to GABA via GABA_A_ receptors. Increased extracellular GABA from inhibited reuptake can activate GABA_A_ receptors, reducing nociception. Notably, other GABA reuptake inhibitors have been reported to produce anti‐nociceptive effects.^64^ Thus, AZ237's ability to influence spiking rates in erythromelalgia‐specific sensory neurons warrants further investigation.

### 4.7 Limitations of the screening method

While this study identified hit compounds with potential mechanisms of action, several limitations exist. The screen relied on sensory neurons differentiated from iPSCs derived from a single donor, necessitating further investigations with a broader range of IEM donors and *SCN9A* mutations. Additionally, variability in spontaneous activity between differentiations is an acknowledged limitation of iPSC‐based models, particularly in ectoderm‐derived lineages such as sensory neurons. This variability can arise from genetic drift during prolonged passaging, subtle differences in differentiation conditions or inter‐batch variation in cell resilience. While we mitigated this by using early‐passage cells from a cryopreserved working stock and synchronised co‐differentiation where possible, cryopreservation of mature sensory neurons remains technically challenging due to axonal coalescence and viability issues. As such, variability remains a recognised constraint in the field and should be considered in the interpretation of functional data.

## 5 CONCLUSION

We developed an MEA‐based approach for screening small molecule compound libraries, identifying molecules that modulate sensory neuron spiking activity and may serve as candidate analgesic drugs. Notably, AZ106 emerged as a potent compound for further investigation as potential treatment for IEM; however, it must be noted that AZ report the possible binding of this compound to multiple receptors other than Na_V_ 1.7 which may complicate its development as a potential analgesic.

## AUTHOR CONTRIBUTIONS

*Experimental design, data acquisition and analysis, figure preparation, manuscript writing*: J. R. T. and A. C. *Data acquisition*: S. H. and T. C. H. *RNA‐Seq analysis*: A. U., F. G. and S. N. G. *Data acquisition and analysis, figure preparation, manuscript writing*: V. T. *Study design, manuscript writing and funding acquisition*: M. L. and E. S. *Study and experimental design, figure preparation, manuscript writing, funding acquisition and overall coordination of the study*: L. A.

## CONFLICT OF INTEREST STATEMENT

The authors declare no conflicts of interest.

### ETHICS STATEMENT

Ethical permission for the use of erythromelalgia patient specific iPSC lines was not required by our institution since these cell lines were purchased from the European Bank for Induced Pluripotent Stem Cells who have already established donor consent under their ethical governance framework.

## Supporting information



Supporting Information

## Data Availability

The data that support the findings of this study are available from the corresponding author upon reasonable request. Some data may not be made available because of privacy or ethical restrictions.
