# Supplementary material for: Screening of candidate analgesics using a patient‐derived human iPSC model of nociception identifies putative compounds for therapeutic treatment
Source: Clin Transl Med. 2025 May 25;15(5):e70339. doi: 10.1002/ctm2.70339 (PMC12104564; doi:10.1002/ctm2.70339)
Supplement: Supplementary file 1 — Supporting Information [file CTM2-15-e70339-s001.docx]

**Supplementary Information**

**Screening of Candidate Analgesics Using a Patient-Derived Human iPSC Model of Nociception Identifies Putative Compounds for Therapeutic Treatment**

Jack R. Thornton ^1^, Alberto Capurro ^1,#^, Sally Harwood ^1^, Thomas C Henderson ^1^, Adrienne Unsworth ^2^, Franziska Görtler ^3^, Sushma Nagaraja-Grellscheid ^3^, Vsevolod Telezhkin ^4^, Majlinda Lako ^1^, Evelyne Sernagor ^1^, Lyle Armstrong ^1*^

1. *Biosciences Institute, Newcastle University, Newcastle-upon-Tyne, United Kingdom*
2. *Bioinformatics Support Unit, Newcastle University, Newcastle-upon-Tyne, United Kingdom*
3. *University of Bergen, Norway*
4. *School of Dental Sciences, Newcastle University, Newcastle-upon-Tyne, United Kingdom*

*# Current Address: Queen Mary University of London, Centre of Neuroscience, Surgery and Trauma. The Blizard Institute, London, United Kingdom*

^*^ Correspondence and reprint requests to L. Armstrong, Biosciences Institute, Newcastle University, International Centre for Life, Newcastle-upon-Tyne, United Kingdom, NE1 7RU; E-mail: [lyle.armstrong@newcastle.ac.uk](mailto:lyle.armstrong@newcastle.ac.uk)

**Author Contributions**

JRT, AC- experimental design, data acquisition and analysis, figure preparation, manuscript writing

SH, TCH – data acquisition

AU, FG, SNG- RNA-Seq analysis

VT, data acquisition and analysis, figure preparation, manuscript writing

ML, ES -study design, manuscript writing, and funding acquisition

LA – study and experimental design, figure preparation, manuscript writing, funding acquisition, and overall coordination of the study.
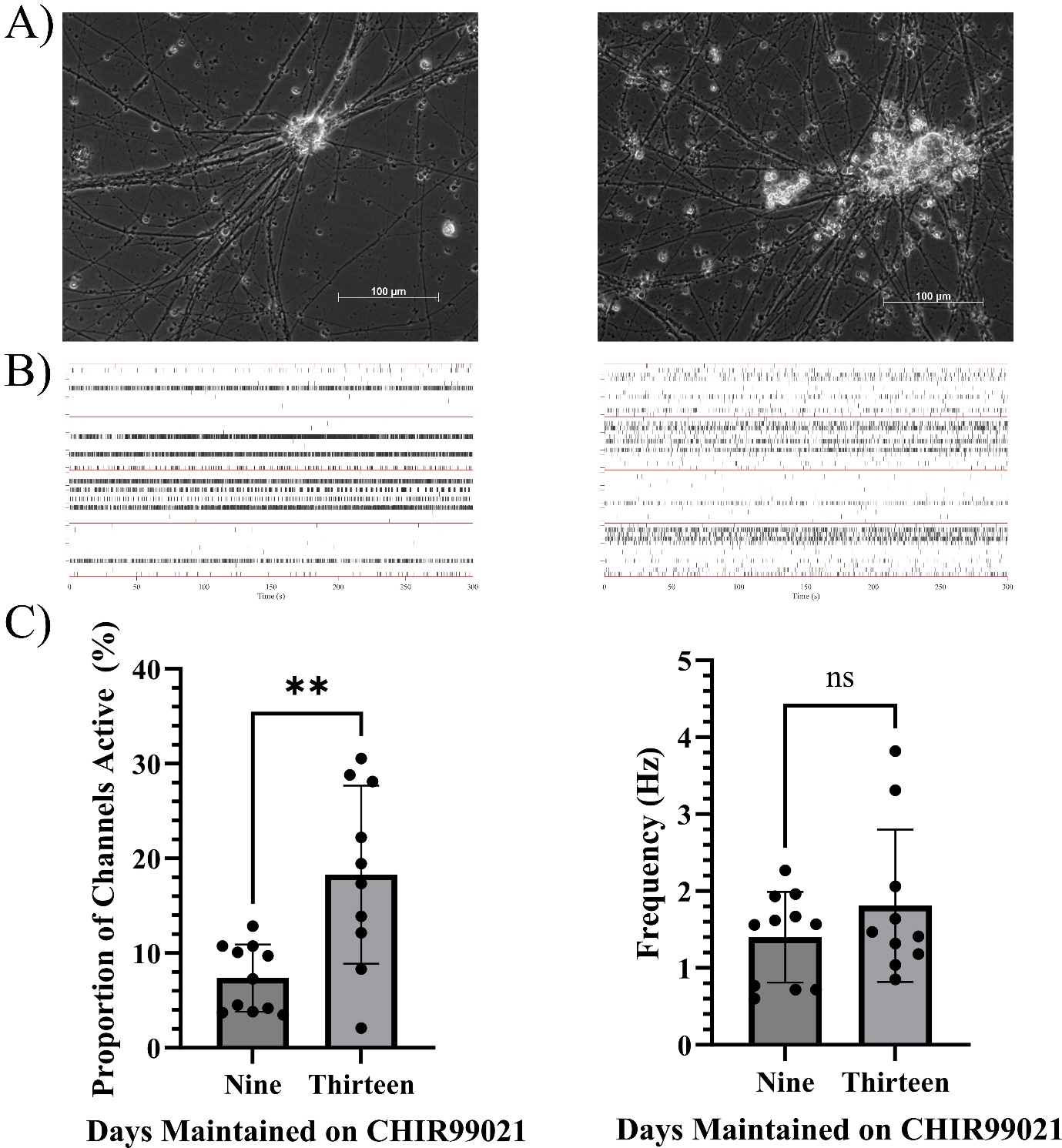


**Figure S1. Comparison of Late-Stage Differentiation in IEM iPSC-derived Sensory neuron-like cells Following CHIR99021 Supplementation.**

A) Representative phase-contrast images of IEM iPSC-derived sensory neuron-like cells cultured with CHIR99021 supplementation for either 9 days (left) or 13 days (right). Morphological differences, including neurite outgrowth and network complexity, are shown. Images were captured at 20 × magnification, with scale bars = 100 μm. B) Raster plots illustrate spontaneous spiking activity in IEM iPSC-derived sensory neuron-like cells following 9-day (left) and 13-day (right) CHIR99021 supplementation, initiating on day 3 and terminating on differentiation days 12 and day 16, respectively. Increased duration of CHIR99021 exposure was associated with noticeable changes in spiking patterns, indicating altered neuronal excitability. C) Quantitative electrophysiological analysis of the proportion of active channels (% of electrodes showing activity) in sensory neuron-like cells with 9-day vs. 13-day CHIR99021 supplementation (left panel) and the mean frequency of spiking activity recorded from active channels in each condition (right panel). Data are presented as mean ± standard SEM, with n ≥ 3 replicates per condition.


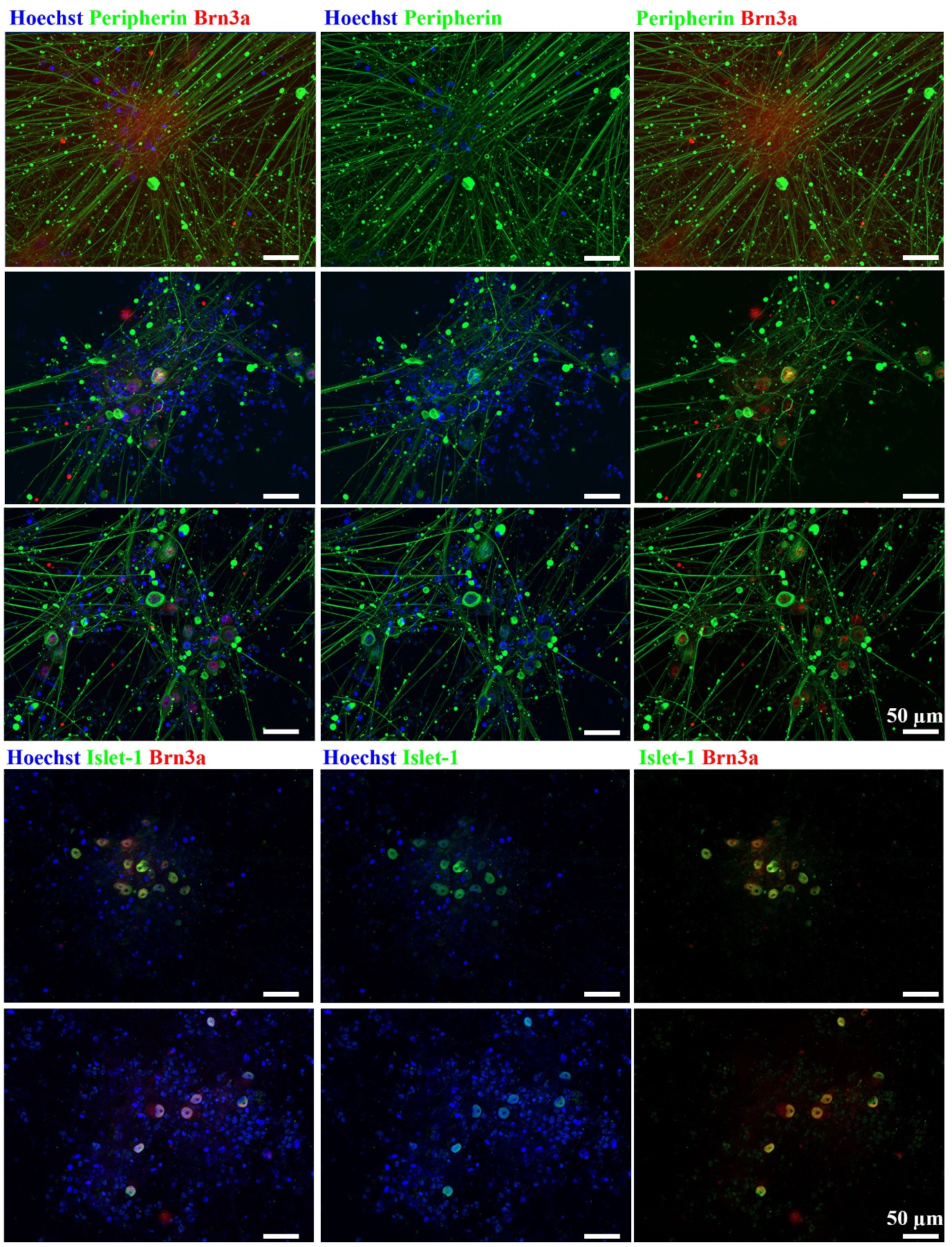


**Figure S2. Immunohistochemical Analysis of Key Proteins in wild type iPSC-Derived sensory neuron-like cells.**

Upper Panels: Representative images showing Brn3a (red) and Peripherin (green) expression in differentiated iPSC-derived sensory neuron-like cells. Staining highlights Brn3a as a marker of neuronal lineage commitment and Peripherin as an indicator of peripheral sensory neuron identity. Lower Panels: Representative images displaying co-expression of Islet-1 (green), a transcription factor involved in sensory neuron development, and Brn3a (red) in the same neuronal cultures. In all panels, Hoechst (blue) was used as a nuclear counterstain to visualise cell nuclei. All images captured at day 70 of differentiation at 20 × magnification, scale bars = 50 μm. The final concentrations for each antibody are shown in **Table S1**.


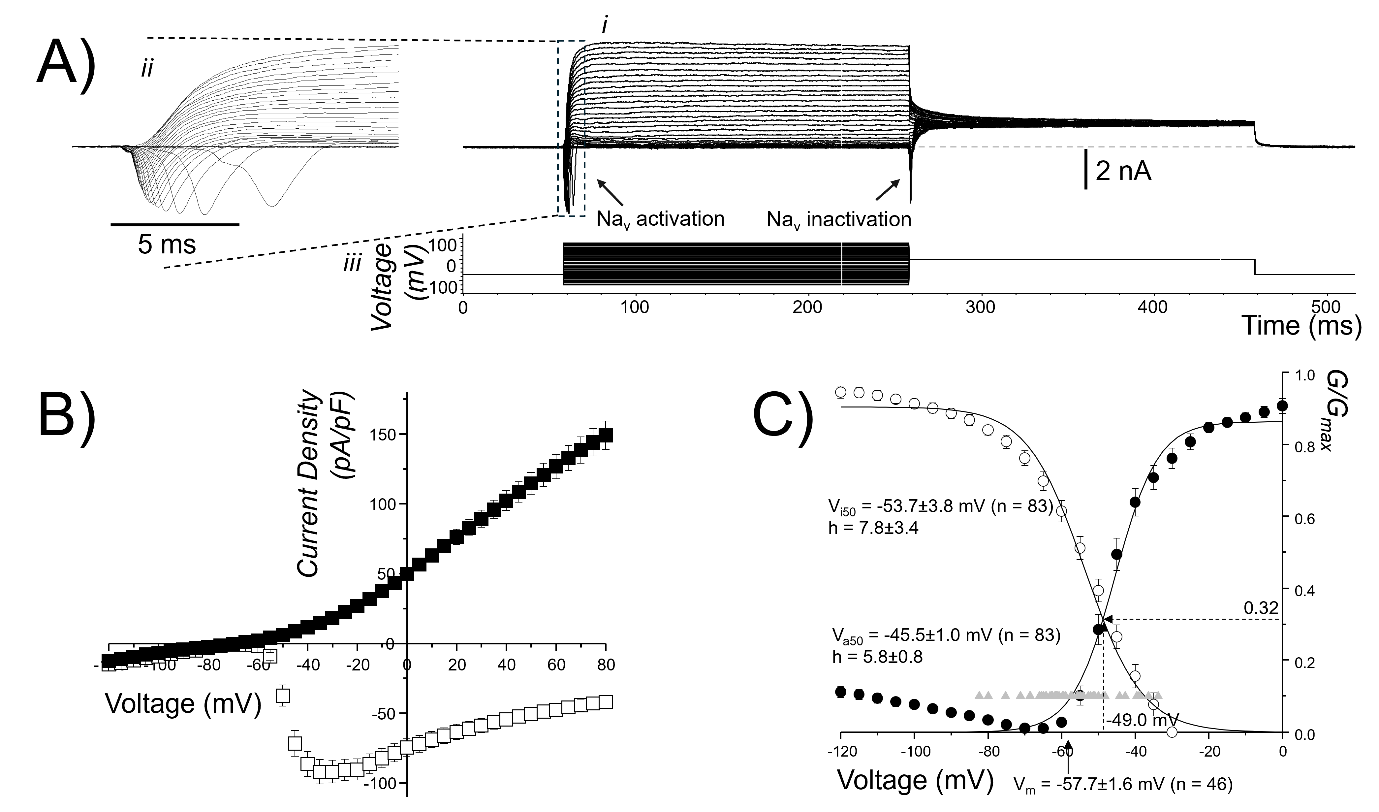


**Figure S3. Macroscopic Na_V_ and K_V_ Currents in IEM iPSC-derived sensory neuron-like cells.**

**A**) Whole-cell macroscopic traces of inward Na^+^ and outward K^+^ currents with activation/inactivation arrows; expanded inset showing Na^+^ currents; voltage-step protocol with holding potential -70 mV, range -120 mV to 80 mV, 5 mV steps. **B**) Na^+^ and K^+^ current densities in IEM- iPSC-derived sensory neuron-like cells. **C**) Normalised Na^+^ conductance activation (filled circles) and inactivation (open circles) curves. Resting membrane potential values shown by grey triangles, availability window maxima and mean V_m_ on x-axis.


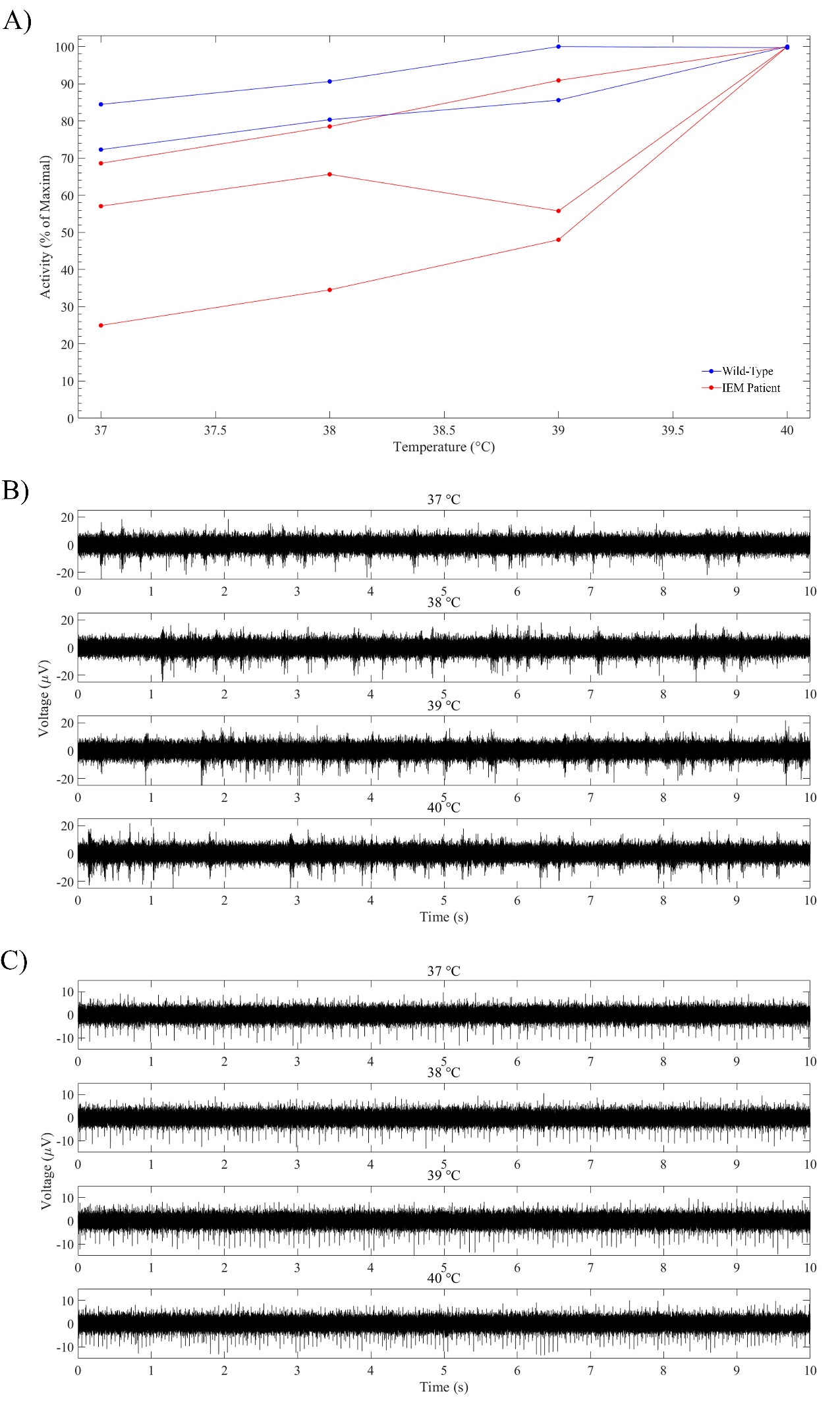


**Figure S4. Impact of Incremental Temperature Increases on Neuronal Activity in IEM and Wild-Type iPSC-Derived sensory neuron-like cells.** Neuronal firing activity was quantified using microelectrode array (MEA) recordings, and responses were normalised to the maximum firing rate observed in control conditions. Data are presented as the percent of maximal firing activity, calculated as (firing rate after treatment / maximal firing rate) × 100, to allow for comparison across conditions and dose-response analysis.

1. Incremental increases in temperature induced a stepwise increase in spontaneous spike activity in sensory neuron-like cells derived from IEM2 (patient-derived) and WT3 (wild-type) iPSCs. Activity was normalised to maximal firing rates recorded across all temperatures to facilitate comparisons between iPSCs-derived sensory neuron-like cells. **B**) Representative raw voltage traces were recorded from IEM2 iPSC-derived sensory neuron-like cells at temperatures of 37° C, 38° C, 39° C, and 40° C. Traces illustrate the gradual increase in spike frequency and neuronal hyperexcitability in response to rising temperatures. **C**) Representative raw voltage traces were recorded from WT3 iPSC-derived sensory neuron-like cells under the same temperature conditions (37° C, 38° C, 39° C, and 40° C). Traces demonstrate a comparatively muted response to temperature increases, reflecting reduced sensitivity to thermal changes in wild-type sensory neuron-like cells. Data are presented as means, with n ≥ 3 replicates per condition. All recordings were conducted using standard MEA techniques described in the methods section.


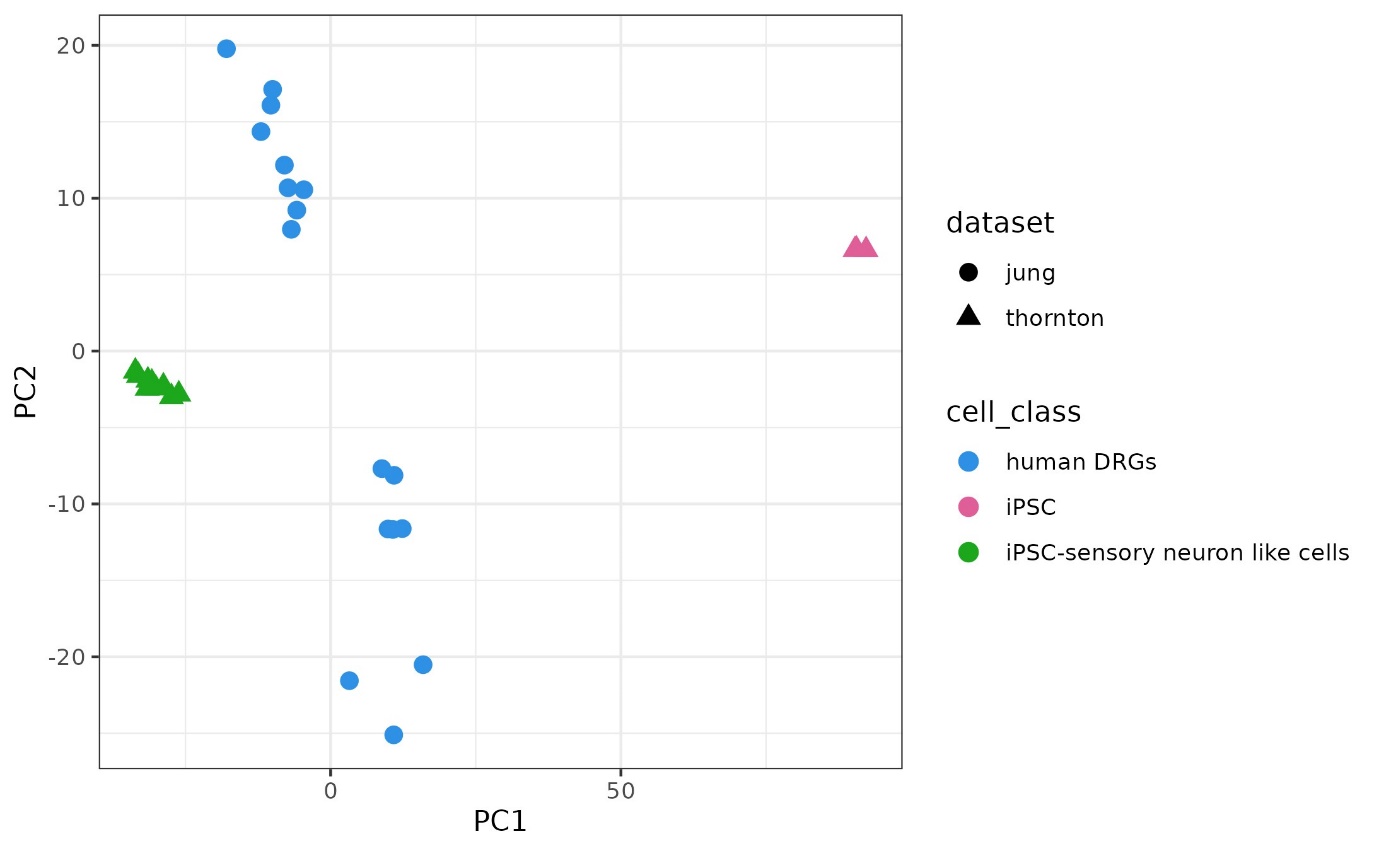


**Figure S5. PCA plot comparing transcriptomic similarities between iPSC-derived sensory neuron-like cells generated in this study and human DRG neurons.** Bulk RNA-Seq from iPSC-derived sensory neuron-like cells generated in this study were compared to a published RNAseq dataset from human DRG neurons (Jung et al [28]). The data from this study (Thornton) are represented by triangles and colour coded for undifferentiated iPSC (pink) or iPSC-derived sensory neuron-like cells (green). The data from the Jung et al study are represented as blue circles and show two independent samples of DRG neurons. It is notable that all samples of iPSC-derived sensory neuron-like cells generated in this study cluster very closely, indicating a high degree of transcriptomic similarity; however, these cells also associate more closely with the clusters of human DRG neurons than they do with the clusters of iPSCs suggesting close similarity between DRG neurons and iPSC-derived sensory neuron-like cells.
